# Multi-AI agent framework reveals the “oxide gatekeeper” in aluminum nanoparticle oxidation

**DOI:** 10.1073/pnas.2537780123

**Published:** 2026-10-15

**Authors:** Yiming Lu, Tingyu Lu, Di Zhang, Lili Ye, Hao Li

**Affiliations:** ^a^ Advanced Institute for Materials Research (WPI-AIMR), Tohoku University, Sendai 980-8577, Japan; ^b^ School of Chemical Engineering, Dalian University of Technology, Dalian, Liaoning 116024, China; ^c^Suzhou MatSource Technology Co., Ltd., Suzhou, Jiangsu 215000, China; ^d^ Frontier Research Institute for Interdisciplinary Sciences, Tohoku University, Sendai 980-8578, Japan

**Keywords:** aluminum nanoparticles, AI agents, machine learning potential, oxidation mechanism

## Abstract

Aluminum nanoparticles (ANPs) are among the most energy-dense solid fuels, yet the atomic mechanisms governing their transition from passivated particles to highly reactive states remain elusive. This stems from a fundamental computational bottleneck: Ab initio methods offer quantum-level fidelity but are restricted to small spatiotemporal scales (<500 atoms, picoseconds), while empirical force fields lack the reactive fidelity required for complex combustion environments. Here, we bridge this gap by employing a human-audited, multiagent framework to make the development and validation of a task-specific machine learning potential (MLP) more traceable and auditable. The workflow coordinates active learning, model evaluation, validation evidence, and human expert review, while leaving scientific interpretation and model-use decisions under researcher control. This collaborative cycle ensures quantum mechanical accuracy while exhibiting near-linear scalability. Strikingly, our simulations reveal a temperature-regulated dual-mode oxidation mechanism: At moderate temperatures, the oxide shell acts as a dynamic “gatekeeper,” regulating oxidation through a “breathing mode” of transient nanochannels; above a critical threshold, a “rupture mode” leads to shell failure and rapid combustion. Importantly, we resolve a decades-old controversy by demonstrating that aluminum cation outward diffusion, rather than oxygen transport, dominates mass transfer in studied regimes, with diffusion coefficients consistently exceeding those of oxygen by 2 to 3 orders of magnitude. These results provide an atomistic framework for interpreting ANP reactivity and illustrate how human-audited agentic workflows can make domain-specific reactive MLP development and deployment more reproducible.

## Significance

Aluminum nanoparticles (ANPs) are premier energetic materials, yet the atomic mechanisms governing their transition from passivated states to highly reactive states remain difficult to resolve. We use a human-audited, multiagent framework to support task-specific MLP development, validation, provenance tracking, and deployment decisions for ANP oxidation simulations. We reveal that the oxide shell functions as a dynamic “gatekeeper,” regulating oxidation through a “breathing mode” at moderate temperatures and a catastrophic “rupture mode” at critical thresholds. The analysis supports Al outward transport as the dominant mass-transfer pathway in the studied regimes. This work provides a validated atomistic framework for interpreting ANP oxidation and illustrates how AI agents can organize MLP development and validation within a human-audited scientific workflow.

The quest for high-performance energy storage and propulsion systems has driven intense interest in aluminum nanoparticles (ANPs), which deliver exceptional energy densities of up to 31 kJ/g (1) and serve as critical components in advanced aerospace and defense technologies (2–4). Despite decades of research, a fundamental mystery has persisted: How do these nanoscale powerhouses transition from benign, passivated particles at ambient conditions to explosive reactants at elevated temperatures? This transition spans room-temperature oxide-shell formation to high-temperature oxidation involving complex gas-phase aluminum species (5–7). The underlying atomic-scale processes that control this transition have remained elusive, creating a critical knowledge gap that limits our ability to precisely engineer energy release rates and optimize combustion efficiency. Resolving this long-standing puzzle requires understanding the mass transport mechanisms that govern ANP reactivity across the targeted temperature spectrum—a challenge that has defied conventional experimental and theoretical approaches due to the extreme spatiotemporal scales involved, from picosecond atomic motions to millisecond macroscopic energy release (8–10).

Accurate simulation of ANP combustion faces a fundamental scientific challenge: achieving large-scale spatiotemporal simulations while maintaining quantum mechanical accuracy. Current mainstream theoretical methods suffer from inherent limitations. Quantum mechanical approaches, exemplified by density functional theory (DFT), can precisely describe interatomic interactions but are computationally prohibitive, restricting simulations to hundreds of atoms and picosecond timescales (11, 12). Conversely, classical reactive force fields (e.g., ReaxFF) can handle larger scales (thousands of atoms, nanosecond timescales) (7, 13) but rely on empirical parameter fitting, which struggles to capture complex charge transfer and structural rearrangements (14). This limitation confines studies to phenomenological observations rather than establishing rigorous physical laws. A critical question arises: Is the previously observed dominance of Al outward diffusion a genuine physical phenomenon, or merely an artifact of parameterization?

To bridge this accuracy-scale gap, active-learning-based machine learning potentials (MLPs) have emerged as a powerful solution (15–17). By learning the potential energy surface (PES) directly from first-principles data, MLPs—such as the attention-based DPA-2 architecture implemented in DeepMD-kit (18, 19), can approach DFT-level accuracy and extend accessible spatiotemporal scales far beyond AIMD, with computational throughput below that of empirical-force-field MD. However, developing a robust reactive MLP for ANP combustion is highly nontrivial. It requires sampling a massive configurational space covering varied bonding environments, interface structures, and transition states. While active learning addresses this by selectively targeting high-uncertainty configurations for DFT labeling (20, 21), it introduces workflow complexities. Researchers must manually manage task planning, threshold tuning, artifact tracking, and validation checkpoints. Relying on ad hoc scripts compromises both the reproducibility of the model and the auditability of its validated domain.

Recent scientific-agent systems have expanded from end-to-end research automation and automated chemical experimentation (22, 23) to domain-specific computational science. In atomistic modeling, MDCrow (24) targets biomolecular molecular dynamics, El Agente (25) supports quantum-chemistry workflows, DREAMS (26) coordinates DFT calculations and HPC-based materials modeling, and ChemGraph (27) provides agent-assisted computational-chemistry workflows. A category-based overview of these and related scientific-agent systems and AI-assisted workflows is provided in *SI Appendix*, Table S3. Together, these studies illustrate how agents can help plan and execute domain-specific scientific tasks. For the reactive-MLP setting considered here, however, the workflow must also maintain dataset and checkpoint lineage, validation evidence, model-use boundaries, and expert control over deployment decisions.

**Fig. 1.**
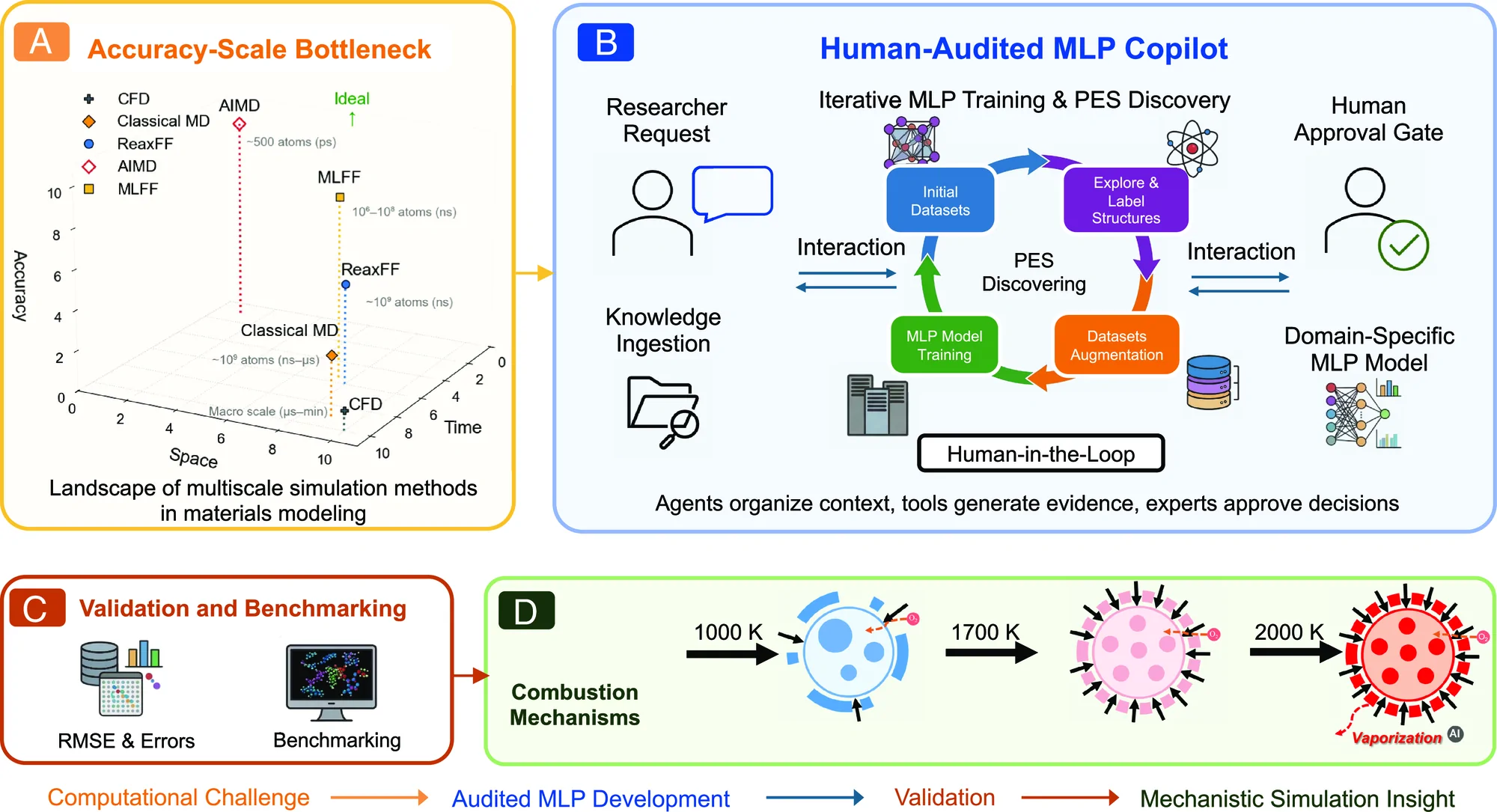
(*A*) Accuracy–scale trade-offs in multiscale simulation methods. (*B*) Human-audited MLP Copilot workflow. (*C*) Model validation and benchmarking. (*D*) ANP oxidation mechanisms at different temperatures. The framework supports task planning, model evaluation, artifact tracking, and human approval checkpoints for MLP deployment, enabling a more organized and auditable route to ANP oxidation simulations beyond DFT-accessible time and length scales (details are provided in *SI Appendix*, Fig. S8).

To meet this need, we developed MLP Copilot, a human-audited multiagent framework that coordinates the reactive MLP lifecycle (Fig. 1). The framework connects task planning, data generation, model evaluation, and retained evidence, while checkpoint acceptance and model-use decisions remain under expert control. Leveraging this framework, we constructed a highly accurate ANP reactive potential and achieved production-scale simulations beyond DFT-accessible scales. Crucially, our simulations resolve the long-standing mechanistic controversy by demonstrating that outward Al diffusion—rather than oxygen transport—dominates mass transfer in both bare and shelled nanoparticles across all studied temperature regimes. Furthermore, we reveal a temperature-regulated dual-mode oxidation mechanism: The oxide shell acts as a dynamic gatekeeper that supports diffusion-limited “breathing” at moderate temperatures and rupture-mediated rapid combustion at elevated temperatures. Together, these results provide an atomic-level framework for ANP reactivity and illustrate how human-audited agentic workflows can make MLP development and deployment more reproducible.

## 1. Results and Discussion

### 1.1. MLP Validation and Dataset Coverage.

**Fig. 2.**
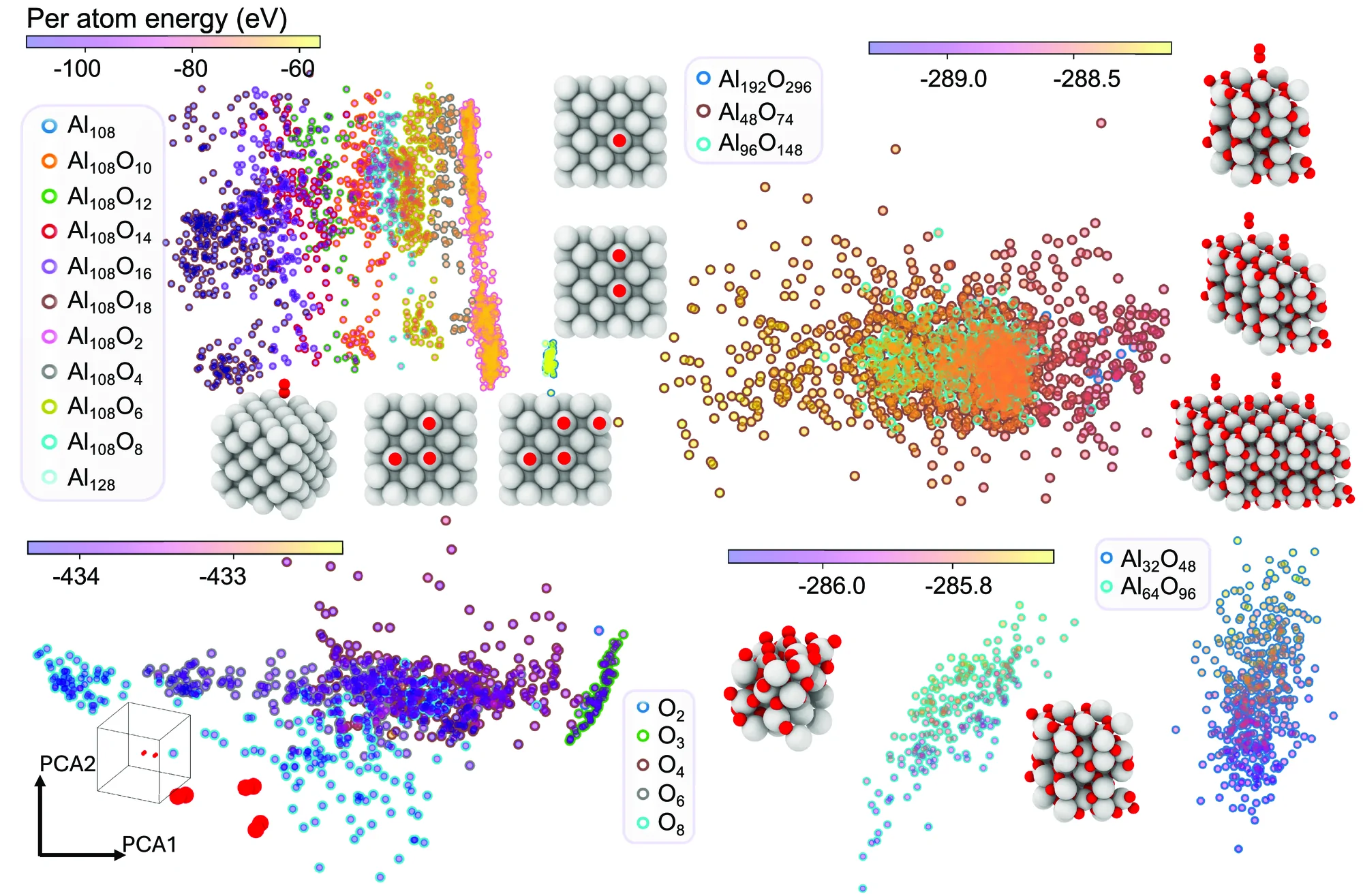
Atomic configuration space mapping and active learning validation based on SOAP. The figure shows the results of dimensionality reduction visualization of 88,833 training configurations through PCA. Each data point represents an independent structure, with its position in the two-dimensional PCA space determined by the first two principal components of SOAP descriptors. The color scale represents per-atom energy, from blue (low-energy) to red (high-energy).

With the assistance of our multi-AI agent framework, accessible via a dedicated frontend interface (see Movie S1 for a demonstration), we employed an iterative active learning strategy incorporating time-stamped force-bias Monte Carlo (tfMC) sampling (detailed in *Methods* and *SI Appendix*) to efficiently explore the Al-O reaction space. After approximately 80 iterations, the resulting dataset contained 88,833 configurations covering temperatures from 200 to 3,000 K. Fig. 2 demonstrates the comprehensiveness of the resulting training dataset within the chemical configuration space. Through principal component analysis (PCA) dimensionality reduction of smooth overlap of atomic positions (SOAP) descriptors (28, 29), we reveal clear structure–energy relationships where configurations with similar chemical environments form distinct clusters. Systems of different stoichiometric ratios occupy different regions, with low-energy equilibrium configurations concentrated at cluster centers and high-energy transition states distributed at cluster edges. This distribution confirms that the active learning strategy successfully captured rare events crucial for ANP combustion simulation, including oxygen diffusion pathways, Al ion migration, and phase transition environments.

Building on this comprehensive dataset, systematic comparison with classical force fields reveals the superior performance of our MLP model (*SI Appendix*, Fig. S2). The model achieves energy Rms error (RMSE) of 1.2 meV/atom and force RMSE of 0.126 eV/Å on testing data, representing 1 to 2 orders of magnitude improvement over traditional potentials. Even for complex nonstoichiometric oxide structures, the model maintains high accuracy while traditional potentials show dramatically increased errors. The force-parity plots and error histograms show predictions centered around the DFT reference, while the global RMSE is calculated over the full high-temperature reactive test distribution, including distorted, undercoordinated, and bond-changing configurations that contribute a long-error tail. Detailed validation results, including structural benchmarks, thermodynamic properties, and phonon calculations, are provided in *SI Appendix*, Figs. S4–S6. The MLP reproduces the phonon dispersion relations and thermodynamic properties (heat capacity and entropy) of Al and Al_2_O_3_ up to 1,000 K. As heat capacity determines the temperature rise rate and entropy correlates with phase transition latent heat, this thermodynamic fidelity, often lacking in classical potentials, is essential for the reliable prediction of combustion dynamics, supporting its use for ANP oxidation simulations within the validated reactive domain.

The target application of the model is nano-scale MD, where validation must address both finite-cluster transferability and the computational cost of applying the model to larger systems. Since direct ab initio molecular dynamics (AIMD) validation remains limited to systems of at most a few hundred atoms, we used isolated, nonperiodic Al_x_O_y_/O_2_ cluster AIMD simulations as an out-of-distribution (OOD) benchmark (*SI Appendix*, Fig. S3). The benchmark spans Al_13_, Al_55_, Al_79_, Al_147_, and Al_309_ clusters, with reported statistics focusing on high-oxygen-coverage reactive subsets. The grouped errors show a clear size dependence: The smallest, most undercoordinated oxygen-rich clusters give the largest deviations, whereas larger nanoparticle-like clusters give lower force and relative-energy RMSEs. For instance, as cluster size increases from Al_13_O_12_ to Al_309_O_12_, the force RMSE decreases from approximately 0.34 to 0.125 eV/Å, and the relative energy RMSE drops from 0.04 to 0.005 eV/atom (*SI Appendix*, Fig. S3*C*). This trend is physically consistent, as larger clusters contain local environments that more closely resemble the nanoparticle surface reactions emphasized in the active-learning dataset. These results confirm that the dataset captures sufficiently diverse local atomic environments for the target ANP oxidation PES, effectively defining the validated domain for subsequent MD simulations. Finally, we evaluated the MD inference cost of the trained MLP beyond the AIMD-accessible scale; the model exhibits near-linear time-per-step and GPU-memory scaling across the tested 100 to 3,000 atom range (details in *SI Appendix*).

### 1.2. Temperature-Dependent ANP Oxidation.

Leveraging the validated MLP model, our large-scale MD simulations establish a unified framework for ANP oxidation that resolves long-standing debates over its governing mechanisms. We reveal a temperature-controlled, dual-mode mechanism where the nanoparticle’s reactivity is ultimately dictated by the structural integrity of its passivating oxide shell. This shell acts as a dynamic gatekeeper, modulating the intrinsically dominant outward migration of Al atoms, which we identify as the fundamental rate-controlling process across all regimes (Movies S2 and S3).

#### 1.2.1. Intrinsic transport mechanisms in bare metal nanoparticles.

**Fig. 3.**
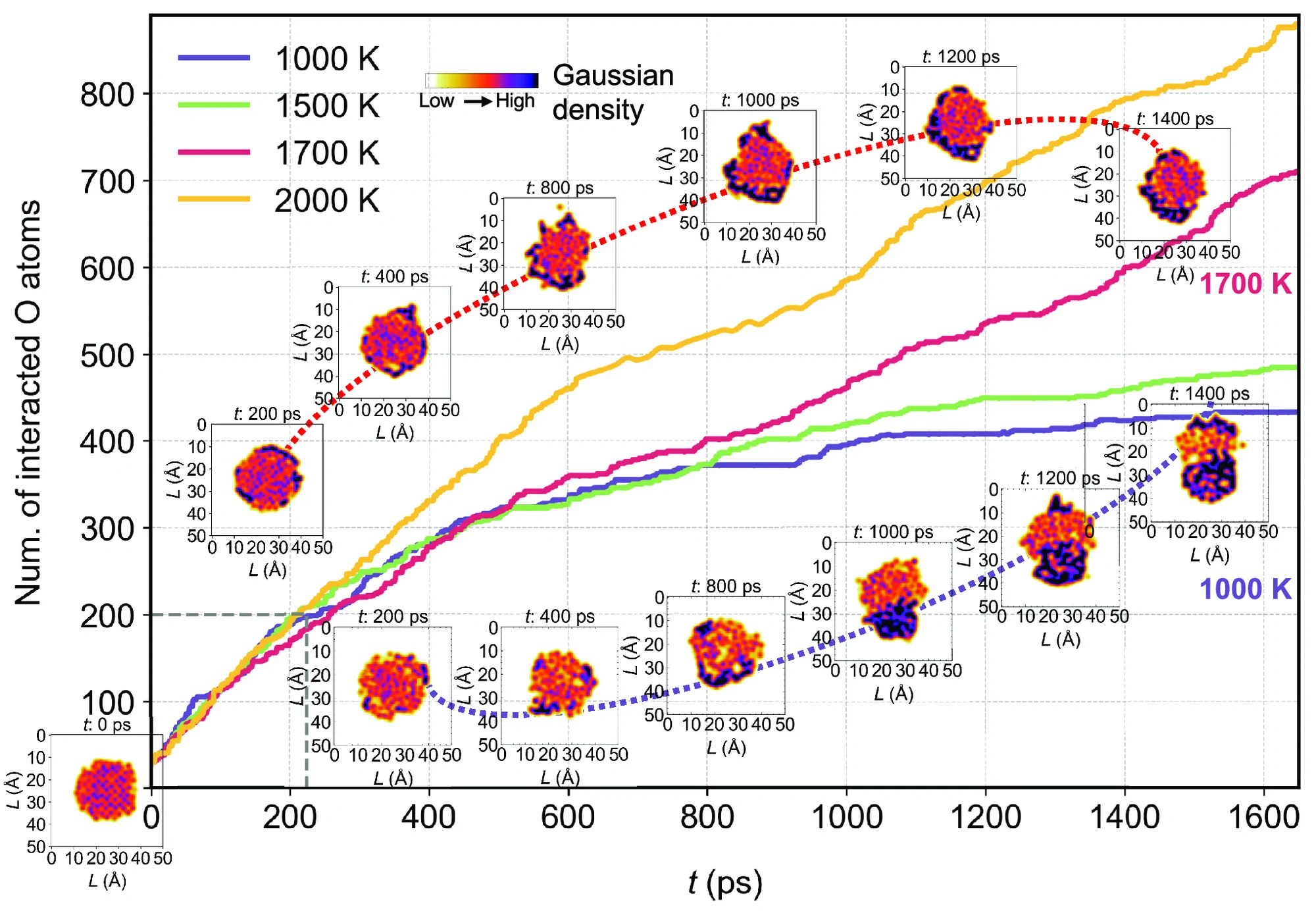
Two-stage oxidation kinetics in bare metal ANP. Initial oxygen-supply limited phase (0 to 200 ps) transitions to temperature-dependent oxidation, with surface passivation at 1,000 K and sustained, more rapid oxidation at higher temperatures. Insets show cross-sectional atomic density distributions.

To uncover the intrinsic oxidation mechanism of ANP, we first investigated bare metal ANPs, isolating the reaction from the complexities of a preexisting oxide shell. Our simulations reveal a universal, two-stage process. Initially, oxidation is limited by the supply of oxygen, with the reaction rate being nearly identical across all studied temperatures (1,000 to 2,000 K) for the first 200 ps (Fig. 3). This temperature-independent behavior confirms that the bare ANP surface is highly reactive, readily consuming all available oxygen and establishing a consistent oxidized state before temperature-specific mechanisms begin to dominate. Following this brief initiation phase, the oxidation pathway diverges into three distinct, temperature-controlled regimes, each characterized by progressively enhanced Al mobility and fundamentally governed by the superior transport properties of Al atoms compared to O. This finding aligns with experimental observations (30) showing that oxygen inward diffusion dominates oxidation at low temperatures (< 600 °C), while Al outward diffusion becomes the primary driving force at high temperatures (>600 °C).

**Fig. 4.**
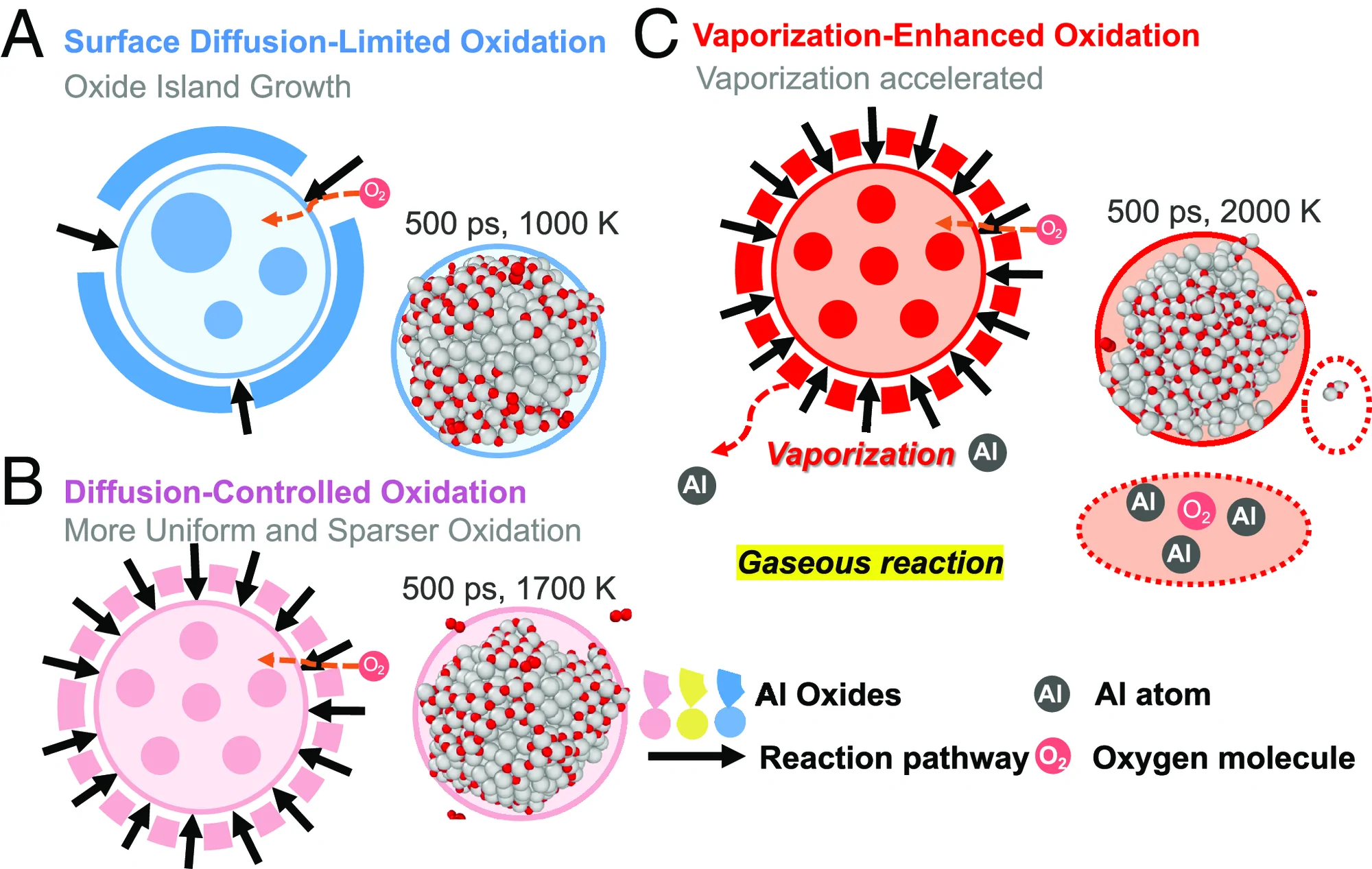
Schematic of temperature-dependent oxidation mechanisms for bare metal ANPs. (*A*) At low temperatures, oxidation is limited by surface diffusion. O atoms have low mobility on the Al surface, leading to the formation of discrete, amorphous oxide islands rather than a uniform shell. (*B*) At intermediate temperatures, enhanced atomic mobility allows for a more uniform but sparser oxide layer to form, representing a diffusion-controlled oxidation regime. (*C*) At high temperatures, oxidation is significantly accelerated by the vaporization of Al atoms from the nanoparticle surface. These vaporized Al atoms react with O_2_ in the gas phase, leading to rapid and complete combustion.

**Fig. 5.**
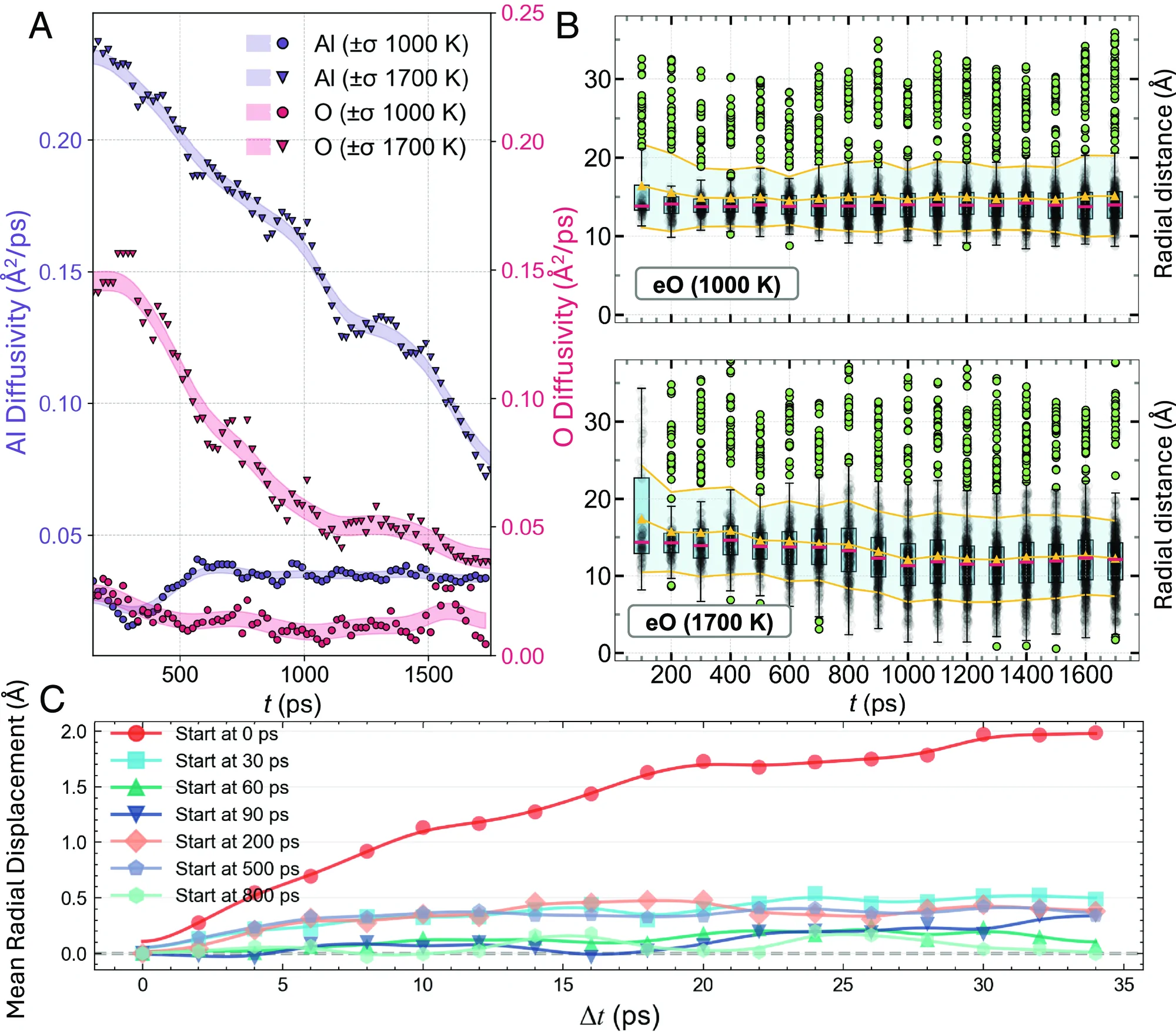
Al-dominated transport mechanisms. (*A*) Time-dependent Al and O diffusivities at 1,000 and 1,700 K show higher Al mobility than oxygen, supporting Al migration as the dominant transport pathway in the studied regimes. (*B*) Radial-distance distributions of initially external oxygen atoms (eO) at 1,000 and 1,700 K track oxygen migration from surface adsorption sites toward the particle interior during oxide growth. (*C*) Direction-resolved cAl cohort analysis reports the mean radial displacement ($\left\langle \Delta r\left(t\right)\right\rangle =\left\langle r_{i}\left(t\right)-r_{i}\left(t_{0}\right)\right\rangle$) for atom identities initialized in the core at selected starting times; positive displacement supports net outward motion of cAl during active oxidation, whereas later cohorts fluctuate closer to zero.

The first temperature regime occurs at low temperature (1,000 K), where oxidation proceeds through a thermodynamically controlled island nucleation and growth mechanism, fundamentally dictated by the kinetic asymmetry between Al and O surface transport. The initial dissociative chemisorption of O_2_ molecules establishes discrete amorphous oxide nuclei (Fig. 4*A*), which subsequently evolves according to classical heterogeneous nucleation theory. The critical mechanistic insight emerges from the pronounced difference in activation barriers: Oxygen adatoms experience severe kinetic trapping with surface migration barriers of 1.25 eV (quantified via PLUMED OPES (31) on Al(100) facets, *SI Appendix*, Fig. S10), rendering them effectively immobile at the available thermal energy (*k*_B_*T* ≈ 0.086 eV). Conversely, Al atoms maintain substantial surface mobility (Fig. 5), while O atoms struggle to diffuse inward as indicated by the barely changed radial distance distribution, creating a dynamic substrate beneath the static oxide patches. To resolve the directionality of Al motion, we further tracked cohorts of initially core Al atoms and computed their mean radial displacement; early-stage cohorts show positive radial displacement, supporting net outward motion of core-origin Al during active oxidation (Fig. 5*C*). This kinetic disparity disfavors uniform film formation and instead drives a preferential edge-growth mechanism, where the high chemical potential gradient at the three-phase boundary (oxide–metal–gas) promotes selective O_2_ adsorption and reaction at island perimeters. The resulting three-dimensional island expansion follows Volmer-Weber growth kinetics until coalescence events progressively reduce the reactive surface area as indicated in Fig. 3. This behavior differs from the chain-like oxide nucleation reported by Zhang et al. using ReaxFF (32), yet both studies corroborate a nucleation-and-growth paradigm in ANP oxidation.

As temperature increases to the intermediate regime (1,700 K), the oxidation mechanism shifts toward more uniform oxide shell growth (Fig. 4*B*), driven by the thermally activated reconstruction dynamics of the molten ANP core. The elevated thermal energy (*k*_B_*T* ≈ 0.146 eV) drives the continuous surface restructuring, effectively disrupting the kinetic stabilization of discrete oxide islands observed at lower temperatures. This dynamic surface reconstruction creates a homogeneous reaction interface that prevents the formation of stable oxide nuclei and overcomes the previously limiting oxygen adatom mobility constraints. The enhanced thermal activation facilitates the predominant migration of Al atoms (Figs. 3 and 5) through a Wagner-type oxidation mechanism, where the chemical potential gradient drives Al diffusion to the reaction front. This Al-dominated mass transport enables the formation of a spatially uniform, continuously growing oxide layer that maintains structural coherence while accommodating volume expansion. The resulting diffusion-controlled oxidation kinetics follow parabolic growth laws, establishing a sustained reaction regime governed by the intrinsic mobility advantage of Al over O in the oxide matrix.

At higher temperatures (>1,700 K), direct Al vaporization emerges as an additional kinetic pathway, consistent with experimental observations at elevated temperature (33). At these elevated thermal conditions (*k*_B_*T* ≥ 0.146 eV), Al atoms acquire more thermal energy to overcome surface binding energies and undergo desorption-mediated mass transport (Fig. 4*C*). This gas-phase reaction pathway alters the reaction kinetics by eliminating the rate-limiting step of solid-state diffusion through the oxide matrix. Quantitative analysis reveals a sharp thermodynamic threshold: While Al atom consumption remains negligible in the moderate temperature range (1,000 to 1,700 K), the desorption flux exhibits exponential temperature dependence, with approximately 40 Al atoms consumed within 1,500 ps at 2,000 K (*SI Appendix*, Fig. S11). The enhanced reactivity manifests through dramatically increased O_2_ consumption rates, where the rapid Al vaporization creates a highly reactive gas-phase environment that efficiently utilizes the replenished oxygen supply. This vaporization-controlled mechanism bypasses the inherent transport limitations of condensed-phase diffusion, establishing a direct gas-phase reaction pathway that accounts for the explosive nature of ANP combustion at high temperatures. The transition represents a shift from transport-limited to thermodynamically driven kinetics, where the reaction rate becomes governed by Al vaporization rather than solid-state diffusion.

Our results on bare nanoparticles reveal that Al-dominated transport is the universal engine of oxidation. However, the reaction’s macroscopic behavior is dictated by temperature, which modulates the mechanism from self-limiting island formation at low temperatures, to steady shell growth at intermediate temperatures, and finally to explosive, vaporization-driven combustion at high temperatures.

#### 1.2.2. Oxide shell gatekeeper effects.

Building on the intrinsic Al-dominated transport mechanism established in bare nanoparticles, we demonstrate that the presence of a preexisting oxide shell introduces a sophisticated “gatekeeper function” that modulates access to reactive sites without altering the fundamental transport physics. This thermodynamically responsive gatekeeper operates through two distinct, temperature-dependent kinetic regimes that represent different manifestations of the same underlying Al transport mechanism under progressively enhanced thermal conditions.

##### 1.2.2.1. Breathing mode.

**Fig. 6.**
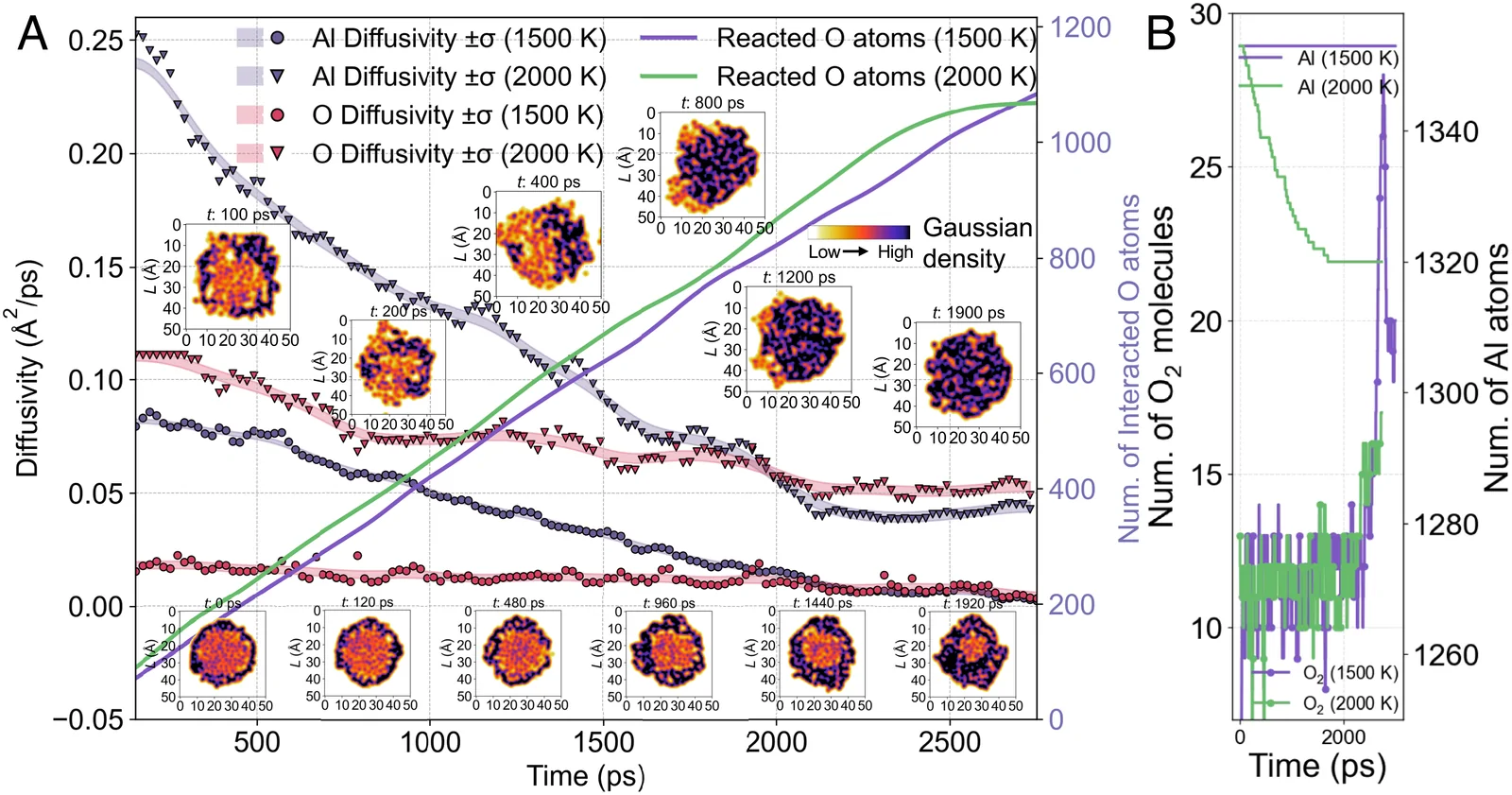
Oxide shell gatekeeper mechanisms modulating Al transport. (*A*) Cross-sectional Gaussian density distributions reveal two distinct modes: “Breathing mode” at 1,500 K with dynamic structural fluctuations creating transient transport channels, and “Rupture mode” at 2,000 K with catastrophic shell failure exposing the reactive core. Diffusion coefficients consistently show Al mobility exceeding oxygen transport, confirming Al-dominated mechanisms persist even with shell constraints (detailed cross-sectional views at different temperatures are shown in *SI Appendix*, Figs. S12 and S13). (*B*) Temporal variation of O_2_ molecules and Al atoms at different temperatures.

In the moderate temperature regime (1,500 K), the oxide shell exhibits thermodynamically controlled structural dynamics characterized by a “breathing mode” mechanism. As demonstrated in Fig. 6*A*, this regime is governed by the interplay between thermal activation energy and shell cohesive forces, resulting in reversible structural fluctuations that generate transient nanoscale transport pathways. Unlike rigid lattice diffusion, these dynamically formed channels function as kinetic bottlenecks (30), establishing a diffusion-limited regime where mass transport across the oxide–metal interface becomes the rate-determining step. The radial distance distribution analysis of different atom groups (*SI Appendix*, Fig. S14) quantitatively confirms the asymmetric transport kinetics: While inward oxygen diffusion proceeds through thermally activated hopping mechanisms with limited penetration depth, outward Al migration continues to dominate but now operates under constrained flux conditions due to the shell’s selective permeability. This temperature-controlled process, mediated by defect-assisted transport through the dynamically “breathing” oxide matrix, establishes a quasi-steady-state oxidation regime characterized by sustained, diffusion-limited kinetics where the shell acts as a molecular sieve. These computational predictions are strongly supported by in situ TEM observations from Zhou et al. (34), where the emergence of surface protrusions and concavities at elevated temperatures (973 to 1,208 K) provides direct experimental evidence of these localized breathing modes and transient transport channels.

##### 1.2.2.2. Rupture mode.

When the temperature exceeds the critical thermodynamic threshold (2,000 K), the system undergoes a catastrophic phase transition to a “shell rupture mode” governed by thermomechanical instability. At this temperature regime, the accumulated thermal stress exceeds the oxide shell’s fracture toughness, triggering rapid structural failure within nanosecond timescales and exposing the highly reactive molten Al core to direct oxidant contact (Fig. 6*A*). This critical transition redefines the reaction’s rate-limiting mechanism, shifting from diffusion-controlled transport within the intact shell to external mass transfer limitations. The shell rupture event dramatically amplifies the system’s reactive surface area and eliminates the primary kinetic barrier, thereby unleashing the intrinsic Al transport potential and enabling direct desorption and gas-phase vaporization (Fig. 6*B*). This mechanistic transition from transport-limited to thermodynamically driven kinetics explains the observed shift to explosive combustion behavior. The experimental counterpart to this catastrophic failure is captured (34), where distinct shell-breaking phenomena and core exposure become prevalent at extreme temperatures (1,373 K), mirroring our simulation results.

Importantly, across both regimes, our analysis consistently shows that the diffusion rate of Al atoms is significantly higher than that of oxygen atoms. This confirms that even in the presence of an oxide shell, outward Al migration remains the dominant mass transport mechanism. Temperature does not change this fundamental fact; instead, it controls the permeability of the oxide shell, which in turn dictates the overall rate of the Al-fed reaction. This critical temperature for rupture is also inversely related to shell thickness (35, 36), suggesting a pathway to tune nanoparticle reactivity by engineering the morphology of the ANP.

#### 1.2.3. Unified temperature-modulated transport framework.

Synthesizing our findings from both bare and core-shell nanoparticles, we arrive at a unified mechanism that reconciles the diverse reactive behaviors of ANPs through a single governing principle: temperature-modulated Al transport controlling reaction site accessibility. This framework confirms that the outwardly diffusing Al flux is the universal engine of oxidation, while the oxide shell acts as a thermodynamic valve. This mechanism operates through the intrinsically superior mobility of Al atoms relative to oxygen, with temperature serving as the critical parameter that determines the structural integrity and permeability of the passivating oxide shell, thereby controlling the number and uniformity of exposed reaction sites available for oxidation.

The mechanism manifests through two distinct kinetic regimes: 1) Diffusion-limited regime, where the intact oxide shell operates in “breathing mode,” creating transient nanochannels that constrain Al flux and maintain controlled oxidation; and 2) Transport-unlimited regime, where thermomechanical shell rupture eliminates diffusion barriers, enabling direct Al vaporization and explosive combustion. This transition represents a continuous evolution of the same fundamental transport process rather than distinct mechanistic pathways.

This unified framework establishes a fundamental design principle for energetic nanomaterials: reactivity control through structural engineering of the oxide shell. The temperature-dependent shell behavior functions as a thermodynamically responsive switch that modulates reaction kinetics by controlling the accessibility of reactive Al sites. This mechanism provides a theoretical foundation for tailoring ANP performance through morphological design parameters (shell thickness, core-shell ratio) and environmental conditions (ambient oxygen concentration, heating rate), enabling predictive control over the transition from controlled energy release to explosive combustion. The universality of Al-dominated transport across all temperature regimes suggests that this principle may extend to other metal-oxide nanoparticle systems, offering broader implications for the rational design of reactive nanomaterials.

## 2. Conclusion

In summary, using MLP Copilot within a human-audited multiagent framework, we developed a reactive-domain MLP model with an active-learning strategy tailored to ANP oxidation. The simulations support a temperature-controlled oxidation mechanism: Bare ANPs evolve from island formation to oxide growth and vaporization as temperature increases, while oxide-shelled ANPs switch from breathing-mode constrained oxidation to rupture-mediated rapid reaction. Across these regimes, the diffusion and radial-cohort analyses support outward Al transport as the dominant mass-transfer process in the studied ANP oxidation environments.

MLP Copilot serves as the central coordination layer of this MLP campaign, linking literature-derived knowledge, active-learning iterations, checkpoint evaluations, OOD validation evidence, provenance records, and human-reviewed decisions. By keeping model evidence, decision context, and retained artifacts within one auditable human-supervised loop, it reduces the fragmentation of conventional script-based MLP development and makes task-specific reactive potentials easier to develop, validate, use, and inspect.

More broadly, this work illustrates how human-audited AI-assisted workflows can support evidence-grounded simulations of reactive materials while keeping checkpoint acceptance, and model use under expert control. Looking forward, we envision that human-audited AI agents will become increasingly valuable collaborators in computational materials discovery.

## 3. Methods

### 3.1. Foundational Dataset Sampling.

Accurate MLPs require comprehensive sampling of the chemical reaction space to represent the diverse local atomic environments that different elements can experience during reactions. For ANP combustion, the training dataset must capture diverse atomic configurations across the reaction potential energy surface, including equilibrium structures of various nanoparticle morphologies, reaction transition states for Al-O bond formation and breaking, interfacial configurations between Al cores and alumina shells, and defect structures that serve as reaction hotspots. Achieving such comprehensive coverage presents a fundamental challenge: Traditional AIMD provides quantum-accurate trajectories but at prohibitive computational cost, severely limiting accessible timescales and configurational diversity, while introducing too much data into the training set. To address this limitation, we adopt a two-stage model training strategy combining initial AIMD sampling with subsequent active learning expansion.

We begin by establishing a foundational dataset through AIMD simulations of bulk Al and Al_2_O_3_ at multiple temperatures using the canonical ensemble with 0.5 fs time steps for 4,000 steps. While this conventional approach cannot adequately sample the vast configurational space required for combustion simulations—particularly the rare events and high-energy transition states that govern reaction kinetics—it provides essential equilibrium structures and thermal fluctuations that serve as a robust foundation for our active learning strategy. This initial dataset becomes the crucial starting point from which the active learning algorithm builds targeted PES coverage of the reactive domain.

### 3.2. Active Learning Implementation.

**Fig. 7.**
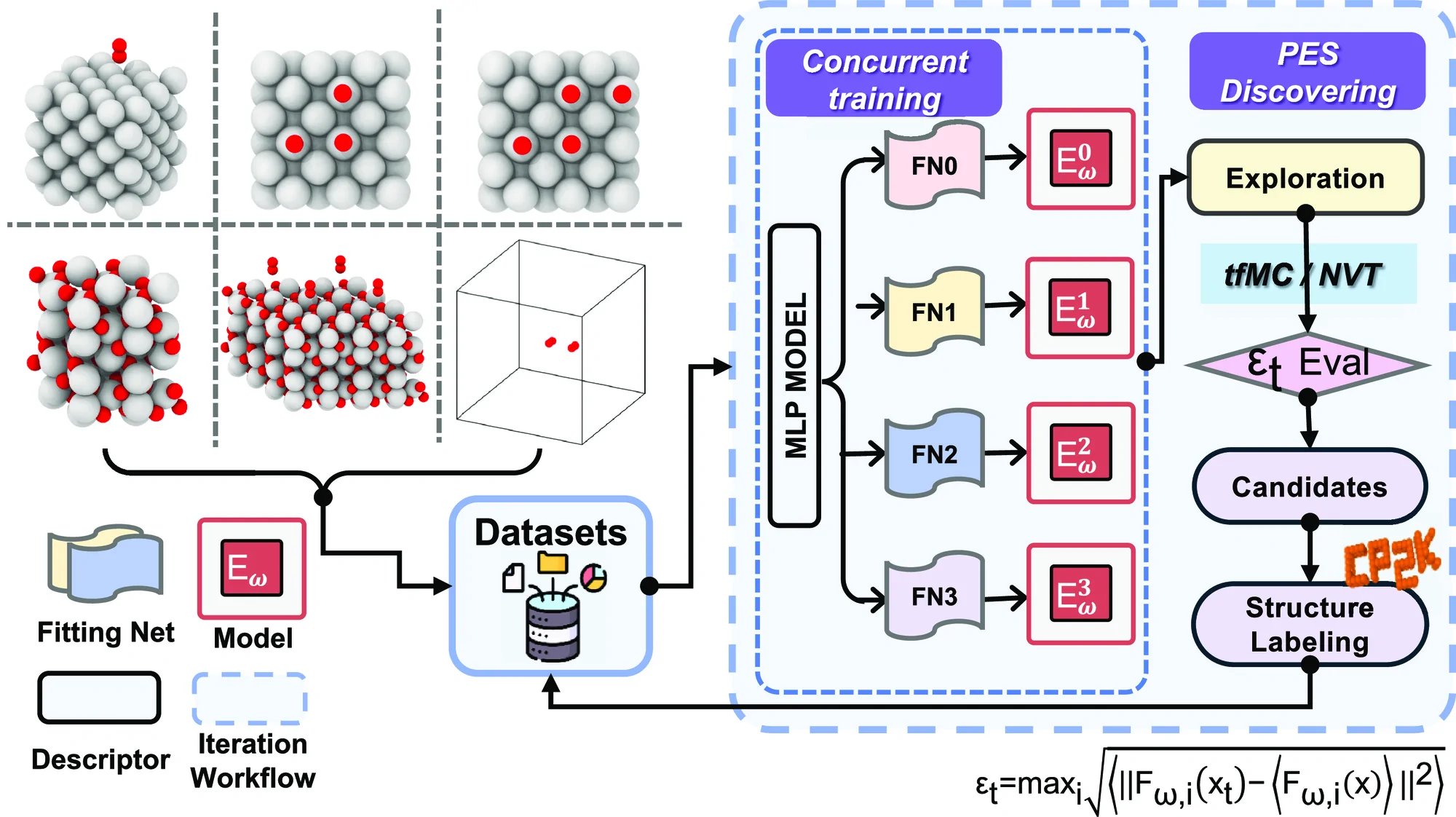
Flowchart of the active learning strategy employed in this study. This cycle enhances the MLP by identifying informative atomic configurations through a Query-by-Committee (QBC) approach. Initially, four concurrent fitting networks (FN0–FN3) with identical hyperparameters but initialized with different random seeds are trained on the existing datasets. During the potential energy surface (PES) discovering phase, the configurational space is explored using tfMC*/*NVT MD. The model evaluates candidate structures by calculating the force deviation ($\varepsilon_{\text{t}}$); structures with uncertainty exceeding a predefined threshold are labeled via CP2K-based DFT calculations and incorporated into the training set. This process repeats until the MLP achieves the desired accuracy across the studied reaction space.

Building upon the foundational dataset established above, our active learning implementation systematically identifies and incorporates the most informative atomic configurations through an iterative approach that progressively enhances model accuracy by focusing computational resources on regions of high uncertainty (Fig. 7). The workflow operates through three integrated stages: ensemble model training, guided exploration using molecular dynamics coupled with tfMC simulation to overcome energy barriers, and uncertainty-based configuration selection for DFT labeling with CP2K (37). This creates an iterative active-learning cycle that systematically expands the model’s predictive scope while ensuring targeted and computationally efficient PES sampling.

To prevent overfitting of MLPs on specific regions of the PES from redundant configurations identified during active learning, we implement a GPU-accelerated structural similarity-based optimization strategy (21) using SOAP descriptors (*SI Appendix*, Fig. S1). This approach, employing a furthest point sampling algorithm, systematically eliminates structurally similar configurations while preserving representative structures that capture essential chemical diversity. By leveraging GPU acceleration for descriptor calculation and clustering, we achieved over 100-fold speed improvements compared to CPU implementations. Detailed parameters for the MLP architecture (*SI Appendix*, Table S1), training protocols, DFT calculations (*SI Appendix*, Table S2), and dataset filtering details are provided in the *SI Appendix*.

### 3.3. Human-Audited Multiagent Framework.

We implemented MLP Copilot, a human-audited multiagent framework, to coordinate active-learning MLP development and validation (*SI Appendix*, Fig. S8). The framework separates executable evidence, agentic coordination, and expert judgment: dataset records, checkpoints, model errors, validation reports, OOD tests, and approval records are produced by tools and retained as file-based artifacts, while language-model agents organize project knowledge, retrieve evidence, assist validation planning, and prepare decision context. They are not treated as independent sources of physical conclusions or model-readiness claims.

Operationally, the workflow links three components: literature and project-context organization through agentic file search; An MLP Copilot runtime that tracks workspace state, dataset paths, checkpoint lineage, validation outputs, artifacts, and approvals; and focused MCP/skill modules for active learning process inspection, dataset validation, checkpoint evaluation, report generation, and evidence collection. DFT settings, MLP framework selection, acceptance criteria, deployment decisions, and mechanistic interpretation remain under expert control. The reusable contribution is the auditable coordination layer: path-based tool access, provenance capture, validation planning, artifact registration, and approval-gated decision support. The CP2K parameters and OOD validation method reported here remain specific to this ANP oxidation study and require rebuilding before transfer to another chemistry regime. Additional implementation details are provided in the *SI Appendix*.

## Supplementary Material

Appendix 01 (PDF)

Movie S1. Demonstration of the DigAuto platform and the multi-agent MLP Copilot workflow. The video showcases the human-audited workflow in which the MLP Copilot supports MLP quality auditing through validation tools and returns evidence for human expert review, enabling a closed-loop feedback mechanism for efficient model development.

Movie S2. Molecular dynamics simulations of the oxidation process of a bare metal aluminum nanoparticle (ANP) at 1000 K and 1700 K.

Movie S3. Molecular dynamics simulations of the combustion process of a core-shell aluminum nanoparticle (Al core with oxide shell) at 1500 K and 2000 K. The video demonstrates the distinct combustion behaviors, capturing the transition from breathing-like oxidation expansion to shell rupture as the temperature increases.

## Data Availability

The MLP Copilot implementation in this study and the comprehensive MLP training dataset are hosted on the Digital Automation for Scientific Discovery platform (DigAuto) at https://www.digauto.org (38). Key computational code, including the MLP Copilot source code, workflow skills, trained MLP models, and analysis scripts, is available in the GitHub repository at https://github.com/flarecentury/mlpcopilot (39).
