# Supplementary material for: Multi-AI agent framework reveals the “oxide gatekeeper” in aluminum nanoparticle oxidation
Source: Proc Natl Acad Sci U S A. 2026 Oct 15;123(42):e2537780123. doi: 10.1073/pnas.2537780123 (PMC13684298; doi:10.1073/pnas.2537780123)
Supplement: Supplementary file 1 [file pnas.2537780123.sapp.pdf]

# Supplementary Information for "Multi-AI Agent Framework Reveals the "Oxide Gatekeeper" in Aluminum Nanoparticle Oxidation"

Yiming Lu[1,2,4], Tingyu Lu[1], Di Zhang[1,3], Lili Ye*[2], Hao Li*[1]

[1] Advanced Institute for Materials Research (WPI-AIMR), Tohoku University, Sendai 980-8577, Japan

[2] School of Chemical Engineering, Dalian University of Technology, Dalian, Liaoning 116024, China

[3] Frontier Research Institute for Interdisciplinary Sciences (FRIS), Tohoku University, Sendai 980-8578, Japan

[4] Suzhou MatSource Technology Co., Ltd., Suzhou 215000, Jiangsu, China

* Corresponding author Email:

yell@dlut.edu.cn (L. Ye)

li.hao.b8@tohoku.ac.jp (H. Li)

## Contents

**Section 1: Machine Learning Potential Development** ........ 2

1.1 Active Learning Dataset Construction ........ 2

1.2 GPU-Accelerated Dataset Simplification ........ 3

1.3 Neural Network Architecture and Training ........ 4

**Section 2: Model Validation and Inference Benchmarking** ........ 5

2.1 In-Domain Accuracy Against DFT ........ 5

2.2 Finite-Cluster Out-of-Distribution Validation ........ 6

2.3 Structural and Thermodynamic Validation ........ 8

2.4 MD Inference Benchmark ........ 10

**Section 3: Human-Audited MLP Copilot Workflow** ........ 11

3.1 Role in Active-Learning MLP Development ........ 12

3.2 Evidence Flow, Provenance, and Approval Gates ........ 12

3.3 Tool Demonstrations and Data Provenance ........ 14

**Section 4: Supplementary Results and Analysis** ........ 15

4.1 Gaussian-Density Analysis of Nanoparticle Morphology ........ 15

4.2 Oxygen-Migration Free-Energy Calculation ........ 15

**Section 5: Supplementary Figures** ........ 16

**Section 6: SI Movie Legends** ........ 20

**References** ........ 20

## Section 1: Machine Learning Potential Development

### 1.1 Active Learning Dataset Construction

We employed an iterative multi-stage active learning strategy to efficiently construct the MLP training set: exploration, uncertainty quantification, selection, and data augmentation. Initial dataset generation involved *ab initio* molecular dynamics (AIMD) simulations of bulk Al and $Al_2O_3$ at multiple temperatures (200 K, 800 K, and 1500 K) using the NVT ensemble with a 0.5 fs timestep for 4000 steps, yielding approximately 24,000 configurations. While the initial dataset diversity was limited due to AIMD sampling inefficiency, it provided a robust foundation for subsequent active learning iterations. We implemented a hierarchical exploration approach, systematically progressing from simple systems (bulk phases and pristine surfaces) at low temperatures to increasingly complex configurations with higher O2 coverage and temperature. This staged methodology includes: (1) initial calculations on bulk Al and O2 for pseudopotential validation, (2) pristine Al(111) surface characterization for baseline electronic structure, and (3) systematic investigation of $O_2$ coverage effects on surface oxide formation. This hierarchical approach ensures comprehensive coverage of the potential energy landscape while maintaining computational efficiency.

Configuration sampling employs a progressive dual-method strategy using the current MLP ensemble trained with different activation functions (Tanh/ReLU). The protocol combines molecular dynamics (MD) and time-stamped force-biasing Monte Carlo (tfMC) simulations [1], [2] to sample thermodynamic conditions from 200 K to 3000 K. Initially, NVT simulations at elevated temperatures accumulate datasets enabling reasonable system descriptions. Subsequently, tfMC simulations (0.16 to 0.23 Å displacement) with increasing step counts (1000–500000 steps) and broader temperature ranges accelerate sampling. Configurations are sampled every 10 to 100 steps, with frequency adjusted based on system reactivity. These approaches are alternately applied based on model performance, creating an adaptive protocol that balances exploration efficiency with model accuracy.

Subsequently, model uncertainty is quantified through the standard deviation of interatomic forces predicted by the MLP ensemble. Configurations with force deviations within adaptive ranges (0.08 to 0.30 eV/Å for low uncertainty; 0.30 to 0.70 eV/Å for high uncertainty) are flagged as "uncertain" and added to a candidate pool. From this pool, configurations are selected for new first-principles calculations using a diversity criterion based on the SOAP descriptor to minimize redundancy.

Finally, selected configurations are incorporated into the training dataset for subsequent MLP training iterations. This iterative process continues until model performance meets predefined criteria. The final dataset comprises approximately 88,833 configurations, ensuring MLP accuracy across the entire relevant configuration space. All configurations were labeled using the CP2K package to obtain corresponding energies and forces, with key parameters summarized in Table S1. The active learning workflow was implemented by adapting the DP-GEN framework [3] with specific code modifications.

**Table S1** First-principles calculation parameters

| Parameter | Value |
|---|---|
| Calculation Method | Gaussian and plane wave (GPW) |
| Exchange-Correlation Functional | PBE + DFT-D3(BJ) |
| Pseudopotentials | GTH |
| Basis Sets | DZVP-MOLOPT-SR-GTH |
| Density Grid Cutoff | 400 Ry |
| Relative Cutoff | 60 Ry |
| Brillouin Zone Sampling | Single Γ point |
| SCF Convergence Criterion | $1\times10^{-5}$ eV |

## 1.2 GPU-Accelerated Dataset Simplification

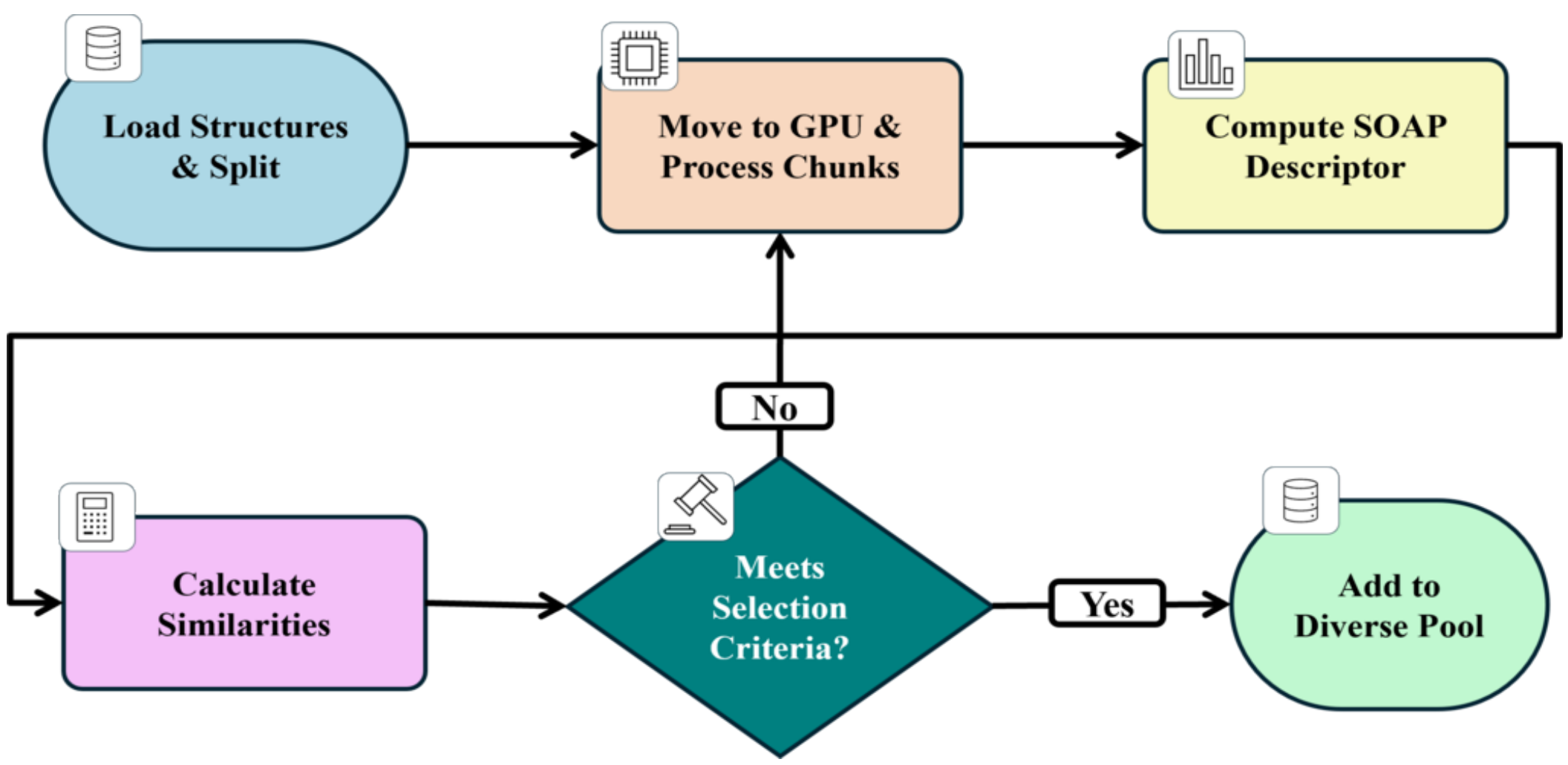


**Fig. S1.** Implementation scheme for structural similarity assessment based on SOAP and CUDA acceleration.

The concurrent learning approach, while effective at identifying high-uncertainty configurations through atomic force variance across multiple models can introduce redundant configurations that provide limited additional training value. This redundancy can lead to overfitting of the MLP on specific regions of the potential energy surface, compromising overall model quality and generalization capability. To address this limitation, we implemented a GPU-accelerated structural similarity-based optimization strategy using SOAP descriptors developed by Zhang et al. [4], achieving over 100-fold speed improvements compared to traditional CPU implementations. The SOAP descriptors capture local atomic environments through spherical harmonics expansion, enabling quantitative assessment of structural similarity across diverse configurations. This approach specifically targets the removal of structurally similar configurations to prevent local overfitting and enhance model performance and generalization capability.

Using the asaplib implementation [5], we systematically eliminate redundant structures while preserving the most representative configurations that capture essential chemical diversity (as shown in Fig. S1). The SOAP hyperparameters are optimized as follows: cutoff radius of 6.8 Å defines the local environment size, $n$=8 radial basis functions control radial resolution, $l$=4 maximum angular degree determines angular resolution, and gaussian width=0.71 Å controls the smoothness of atomic density representation. The similarity calculation employs threshold parameters $S_{max}$=$S_{ave}$=0.06 to identify and remove structurally redundant configurations [4]. The structural similarity assessment is implemented through efficient vectorized distance calculations, where for two sets of SOAP descriptors $\mathbf{A} \in \mathbb{R}^{N_A \times d}$ (candidate structures) and $\mathbf{B} \in \mathbb{R}^{N_B \times d}$ (training structures), the Euclidean distance between descriptors $\mathbf{A}_i$ and $\mathbf{B}_j$ is computed as:

$$d_{ij} = \sqrt{\| \mathbf{B}_j \|^2 + \| \mathbf{A}_i \|^2 - 2\mathbf{B}_j \cdot \mathbf{A}_i}$$

For each training structure descriptor $\mathbf{B}_j$, the minimum distance to any candidate structure is calculated as $D_j^{\min} = \min_{i=1,\dots,N_A} d_{ij}$, and the structural diversity metrics are defined as the maximum and average minimum distances:

$$D_{\max} = \max_{j=1,\dots,N_B} D_j^{\min}, \quad D_{\text{avg}} = \frac{1}{N_B} \sum_{j=1}^{N_B} D_j^{\min}$$

This vectorized implementation leverages GPU parallelization to efficiently process large datasets, with the distance matrix computation optimized through broadcast operations and memory-efficient algorithms, enabling fast structural filtering during active learning iterations.

## 1.3 Neural Network Architecture and Training

The MLP is based on the DPA-2 architecture [6], [7], utilizing a smooth, rotationally invariant descriptor to characterize local atomic environments with a cutoff radius of 6.0 Å and smoothing starting at 5.5 Å. The neural network comprises two primary components: an embedding network and a fitting network. The embedding network maps local environment descriptors to fixed-dimensional representations through three hidden layers containing 25, 50, and 100 neurons, respectively. The fitting network predicts atomic energy contributions *via* three hidden layers, each containing 240 neurons. Model training utilizes the Adam optimizer with an initial learning rate of 0.001, which decays exponentially to $3.51\times10^{-8}$ over 300,000 training steps, applying a decay rate of 0.95 every 1250 steps. The loss function combines energy and force errors as a weighted sum:

$$L = \lambda_E \times \text{MSE}(E) + \lambda_F \times \text{MSE}(F)$$

where $\lambda_E$ and $\lambda_F$ are the relative weights for energy and force contributions, respectively. The weights of energy and force terms used in the training start from 0.02:1000 and gradually converge to 1:1. These weights are chosen to ensure that force accuracy, which is crucial for molecular dynamics simulations, is prioritized while maintaining reasonable energy predictions. The dataset was partitioned into training, validation, and test sets with a 8:1:1 ratio. Training

employed early stopping based on validation set performance to prevent overfitting, with detailed training parameters summarized in Table S2.

**Table S2** MLP training parameters used in this work for Al-O system

| Module | Parameter | Value | Description |
|---|---|---|---|
| **Repinit** | rcut | 8.0 Å | Cutoff radius |
| | nsel | 120 | Maximum neighbor selection |
| | neuron | [25, 50, 100] | Embedding network neurons |
| | three_body_rcut | 4.0 Å | Three-body cutoff radius |
| **Repformer** | rcut | 6.0 Å | Cutoff radius |
| | nsel | 40 | Maximum neighbor selection |
| | nlayers | 6 | Number of layers |
| | g1_dim | 128 | Single-body descriptor dimension |
| | g2_dim | 32 | Two-body descriptor dimension |
| **Fitting Network** | neuron | [240, 240, 240] | Fitting network neurons |
| | activation_function | Tanh/Relu | Activation function |
| | resnet_dt | true | ResNet time step |
| **Training** | numb_steps | 300,000 | Training steps |
| | batch_size | 1 | Batch size |
| | start_lr | 0.001 | Initial learning rate |
| | stop_lr | $3.51\times10^{-8}$ | Final learning rate |
| | decay_steps | 1,250 | Learning rate decay steps |
| | decay_rate | 0.95 | Learning rate decay rate |

## Section 2: Model Validation and Inference Benchmarking

### 2.1 In-Domain Accuracy Against DFT

The held-out test set evaluates the final model over the reactive Al/O configuration distribution generated by the active-learning workflow. On the test set, the final model achieved root-mean-square errors (RMSEs) of 1.2 meV/atom for energy and 0.126 eV/Å for forces (Fig. S2). Compared with classical force fields (EAM [8], ReaxFF [9]), our MLP demonstrates several orders of magnitude improvement, particularly in force prediction accuracy, showcasing excellent predictive performance across the targeted configuration space.

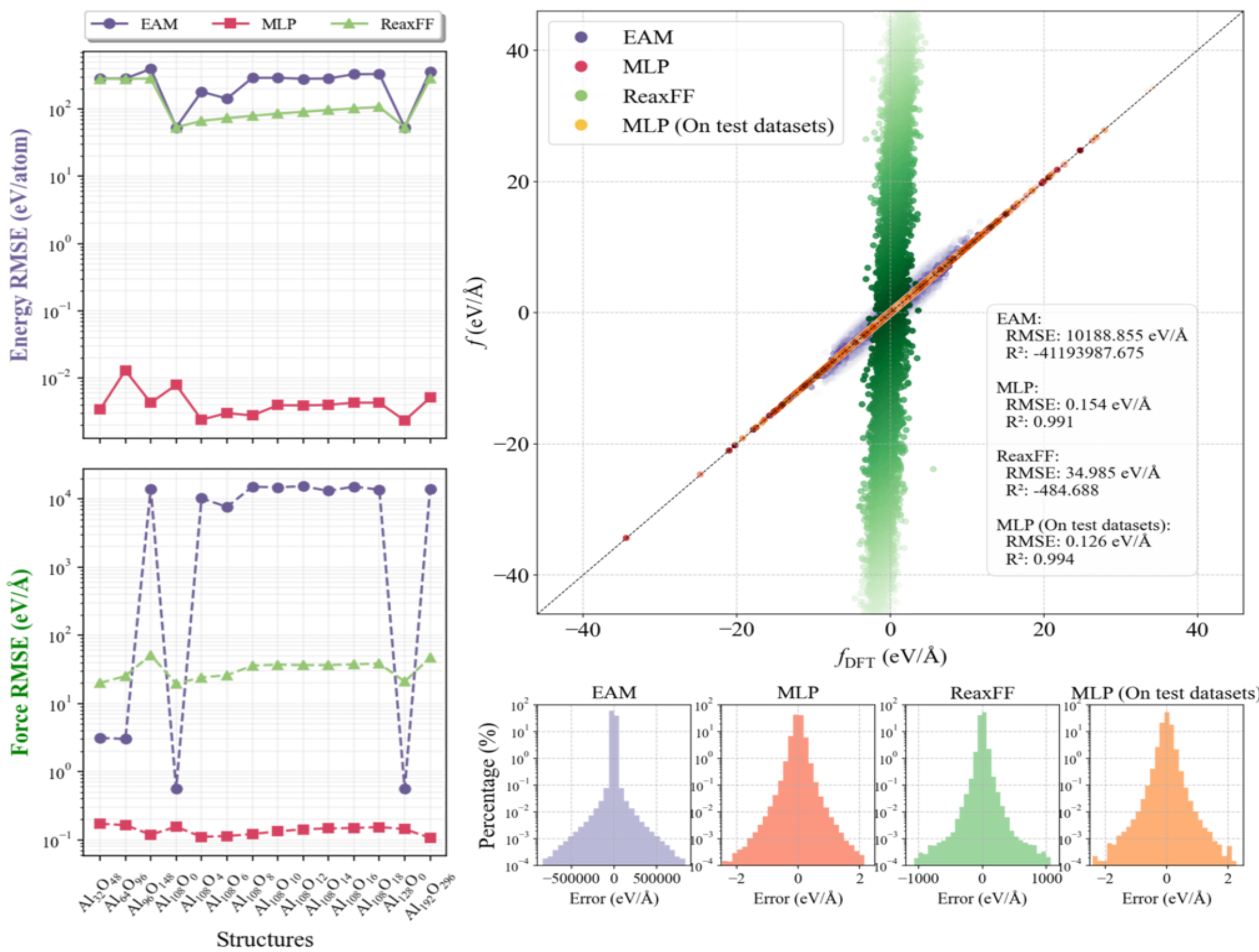


**Fig. S2.** Accuracy evaluation of MLP versus classical force fields. Left panel shows RMSE comparison for energy and force predictions across various aluminum oxide systems. Right panel presents scatter plots and error distribution histograms comparing MLP and ReaxFF predictions against DFT benchmark values for energy and force on the test set.

We omit direct numerical comparisons with other MLPs or foundation models primarily because our potential was developed through an active-learning workflow. Active learning explicitly expands the dataset by sampling configurations where the current model struggles. This yields a training distribution intrinsically tailored to the weaknesses of this specific architecture, rather than a universal benchmark. Evaluating other models on this architecture-biased dataset without matched partitions would be inherently unfair. Instead of head-to-head comparisons, we turned to rigorous out-of-distribution (OOD) validation to demonstrate our model's robustness and physical transferability.

## 2.2 Finite-Cluster Out-of-Distribution Validation

Specifically, we constructed an OOD benchmark using isolated, non-periodic $Al_x/O_2$ cluster AIMD trajectories to test the model's ability to transfer from periodic local environments to finite, cluster-like configurations. These trajectories were generated in CP2K using non-periodic boundary conditions and the WAVELET Poisson solver. The main active-learning dataset was generated primarily from periodic bulk, surface, and reactive-cell calculations, and the production MLP-MD simulations shown in Figs. 3 and 6 used periodic simulation cells. In contrast, the OOD cluster AIMD benchmark in Fig. S3 was deliberately computed without

periodic boundary conditions to test transfer to isolated finite clusters. We simulated $Al_{13}$, $Al_{55}$, $Al_{79}$, $Al_{147}$, and $Al_{309}$ clusters with $O_2$ molecules initially placed near the cluster surface at 700 and 1200 K.

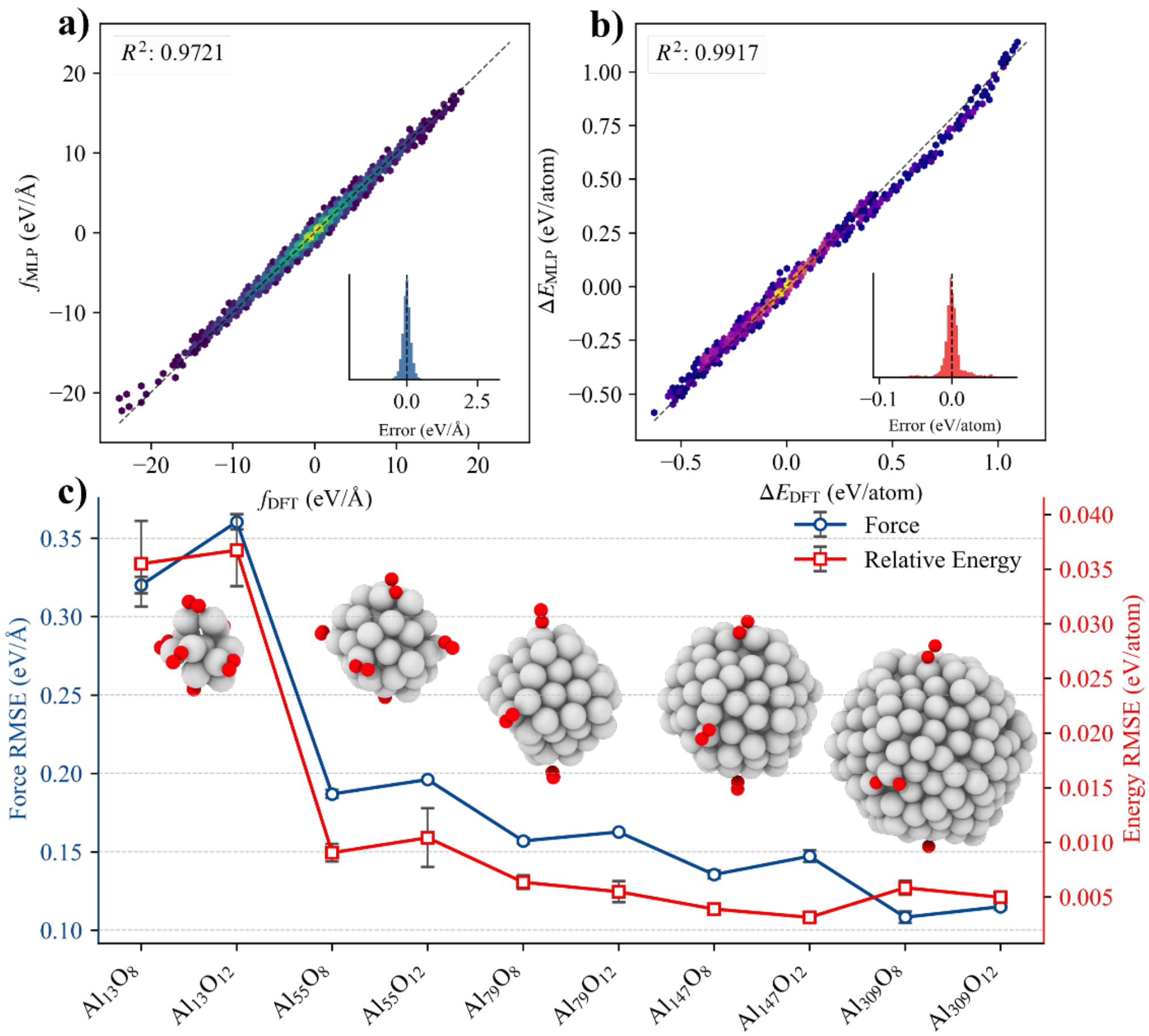


**Fig. S3**. OOD validation on isolated, non-periodic Alx/$O_2$ clusters. (a) Force parity compares reference and MLP force components over retained atoms, frames, with the inset shows the force-error distribution. (b) Relative-energy parity, each point is one retained configuration, and energies are reported per atom after subtracting the mean energy of the corresponding AIMD simulation. (c) Grouped force and relative-energy RMSE values for $Al_{13}$, $Al_{55}$, $Al_{79}$, $Al_{147}$, and $Al_{309}$ configurations.

For the highly reactive, high-oxygen-coverage subset (detailed in Fig. S3), each point in the displayed energy-parity panel represents one retained configuration. Energies are reported per atom after subtracting the mean energy of the corresponding AIMD simulation, yielding an excellent overall correlation ($R^2$ = 0.9911). The corresponding OOD force comparison gives an overall force RMSE of 0.155 eV/Å with $R^2$ = 0.9721. Importantly, the grouped errors reveal a strong size dependence. Force errors are largest for the smallest, most under-coordinated clusters (e.g., ~0.34 eV/Å for $Al_{13}O_{12}$) and systematically decrease toward larger, nanoparticle-like clusters (e.g., ~0.125 eV/Å for $Al_{309}O_{12}$). The grouped relative-energy RMSE similarly summarizes these mean-centered energy deviations, dropping from 0.04 to 0.005 eV/atom across the same size range. This benchmark comprehensively supports the model's finite-cluster transferability within the tested ANP oxidation domain.

## 2.3 Structural and Thermodynamic Validation

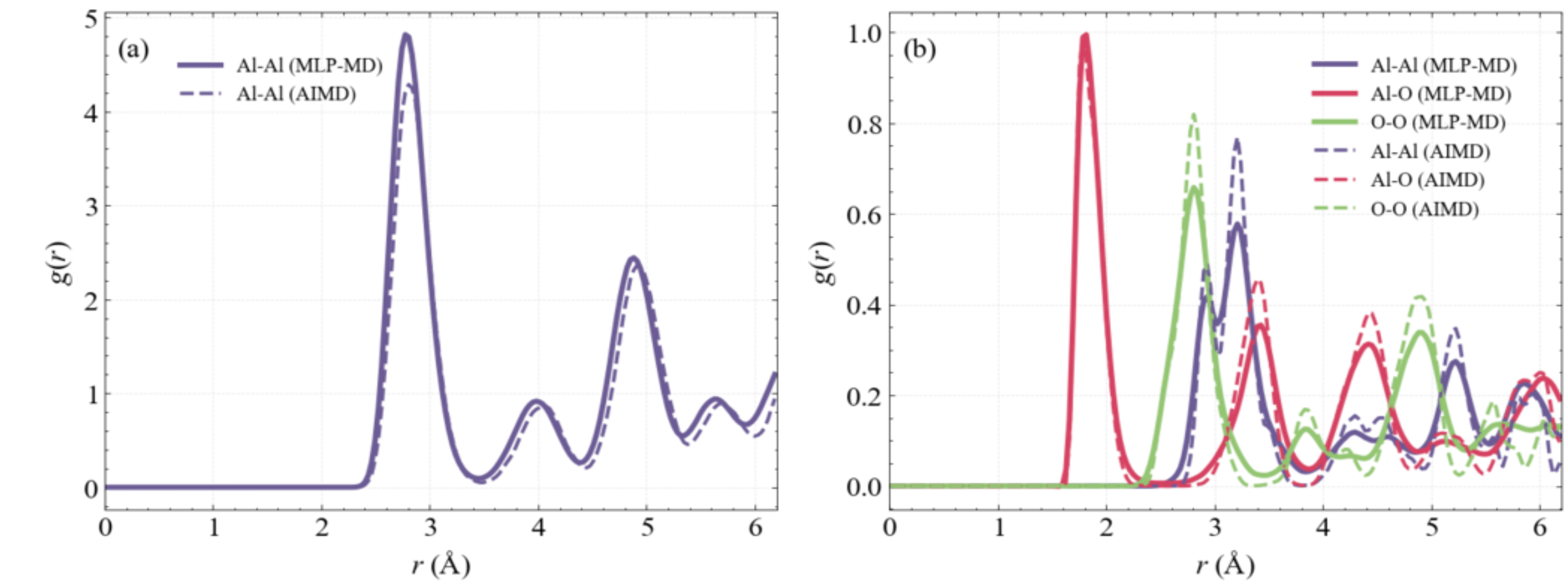


**Fig. S4.** Structural validation based on RDF: comparison of MLP and AIMD simulation results for (a) bulk aluminum and (b) aluminum oxide.

Radial distribution functions (RDF) serve as the "gold standard" for examining structural accuracy in atomic simulations. As shown in Fig. S4, for pure aluminum, the Al-Al RDF calculated by the MLP is in excellent agreement with ab AIMD benchmarks and reference data from an MLP trained specifically for a pure Al system [10], confirming the MLP's precise description of metallic bonding. In the more complex $Al_2O_3$ system, the MLP accurately reproduces the partial radial distribution functions for all three atomic pairs (Al-Al, Al-O, and O-O), demonstrating its ability to simultaneously handle the complex interactions of metallic, ionic, and covalent bonds. This structural fidelity is crucial for combustion simulation, as accurate description of Al-O interactions is central to modeling oxide layer formation and evolution, while precise capture of short-range ordered structures ensures the reliability of key processes such as oxygen atom diffusion and interfacial reactions.

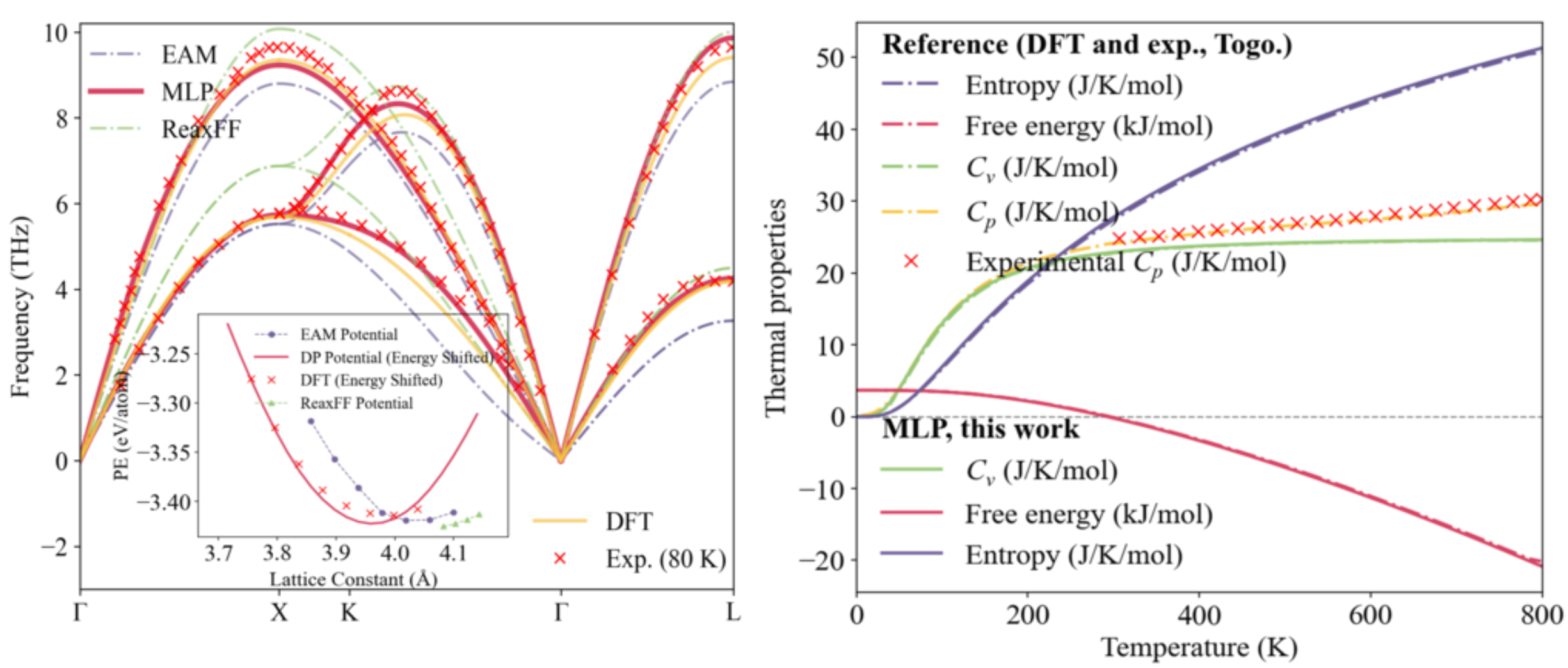


**Fig. S5.** Left panel: Bulk aluminum property comparison (FCC), including (a) equation of state and (b) phonon dispersion curves, comparing MLP, EAM, ReaxFF with DFT and experimental

benchmarks. Right panel: Temperature-dependent thermodynamic properties of bulk aluminum, including Helmholtz free energy, heat capacity, and entropy.

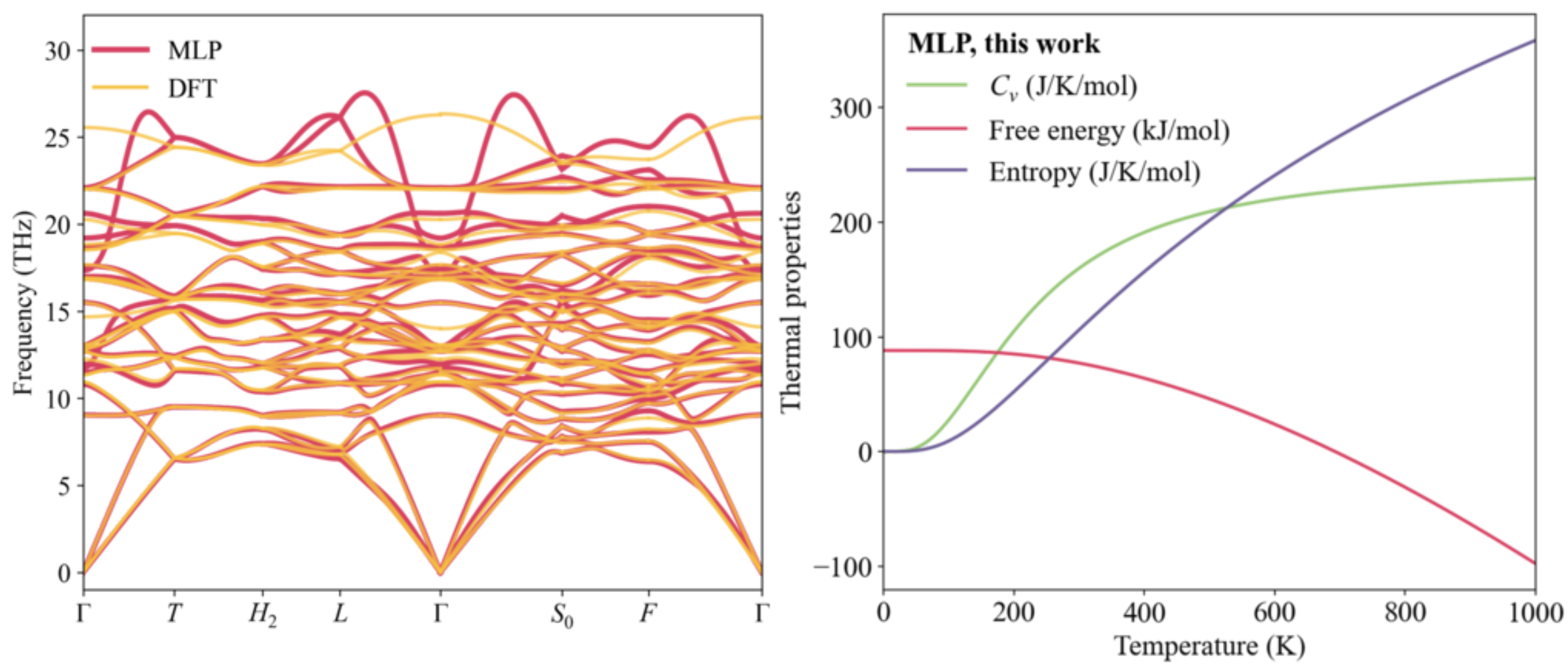


**Fig. S6.** Left panel: Phonon dispersion curves of bulk $Al_2O_3$, comparing MLP with DFT benchmarks. Right panel: Temperature-dependent thermodynamic properties of bulk $Al_2O_3$, including Helmholtz free energy, heat capacity, and entropy.

Equations of state and phonon spectra are core standards for evaluating a potential's quality, reflecting its accuracy in describing static energy and atomic dynamics, respectively. This study utilized the mdapy library [11] to calculate the phonon frequencies of bulk aluminum *via* the finite displacement method (Fig. S5). The results demonstrate that the MLP predicted energy-volume curve agrees well with DFT benchmarks. Although not as accurate as an MLP trained exclusively for a pure Al system [12], [13], this level of precision is acceptable considering the highly complex potential energy surface required for studying ANP combustion. The MLP accurately predicts equilibrium lattice constants and bulk moduli, whereas traditional EAM and ReaxFF potentials exhibit obvious deviations. In the phonon dispersion relations, the MLP model is highly consistent with both DFT calculations and experimental data at 80 K, accurately reproducing the acoustic branches throughout the entire Brillouin zone, while traditional potentials show significant differences in the medium-to-high frequency regions [14].

Additionally, phonon spectrum comparisons for $Al_2O_3$ show that except for the high-frequency region near the gamma point, MLP predictions almost completely overlap with DFT calculation results (Fig. S6). Accurate phonon spectra ensure the reliability of thermodynamic properties such as thermal transport and heat capacity, which are crucial for simulating heat transfer during combustion processes. Accurate equations of state guarantee the credibility of material behavior simulation under high-pressure environments. The excellent performance of the MLP model in these two fundamental physical properties establishes a solid foundation for large-scale, long-timescale combustion dynamics simulations.

Thermodynamic property calculations based on phonon spectra provide final validation for the MLP model (Fig. S5 and S6). Taking Al as an example, within the 0 to 1000 K temperature range, the MLP model's predictions of Helmholtz free energy, constant-volume heat capacity, and entropy are highly consistent with DFT benchmarks and accurately reproduce the physical

laws of the Debye model: $C_v$ increases with $T^3$ in the low-temperature region and approaches the Dulong-Petit limit at high temperatures. In contrast, EAM and ReaxFF calculations show incorrect temperature-dependent behavior, with ReaxFF's heat capacity predictions at high temperatures severely deviating. Accurate prediction of these thermodynamic properties is crucial for combustion simulation, as accurate heat capacity is key to calculating material temperature rise, while precise entropy ensures the reliability of phase transition latent heat. The excellent performance of the MLP model in these fundamental thermodynamic quantities, combined with its successful validation in structural and dynamic properties, comprehensively establishes the credibility of this model for studying complex combustion phenomena.

## 2.4 MD Inference Benchmark

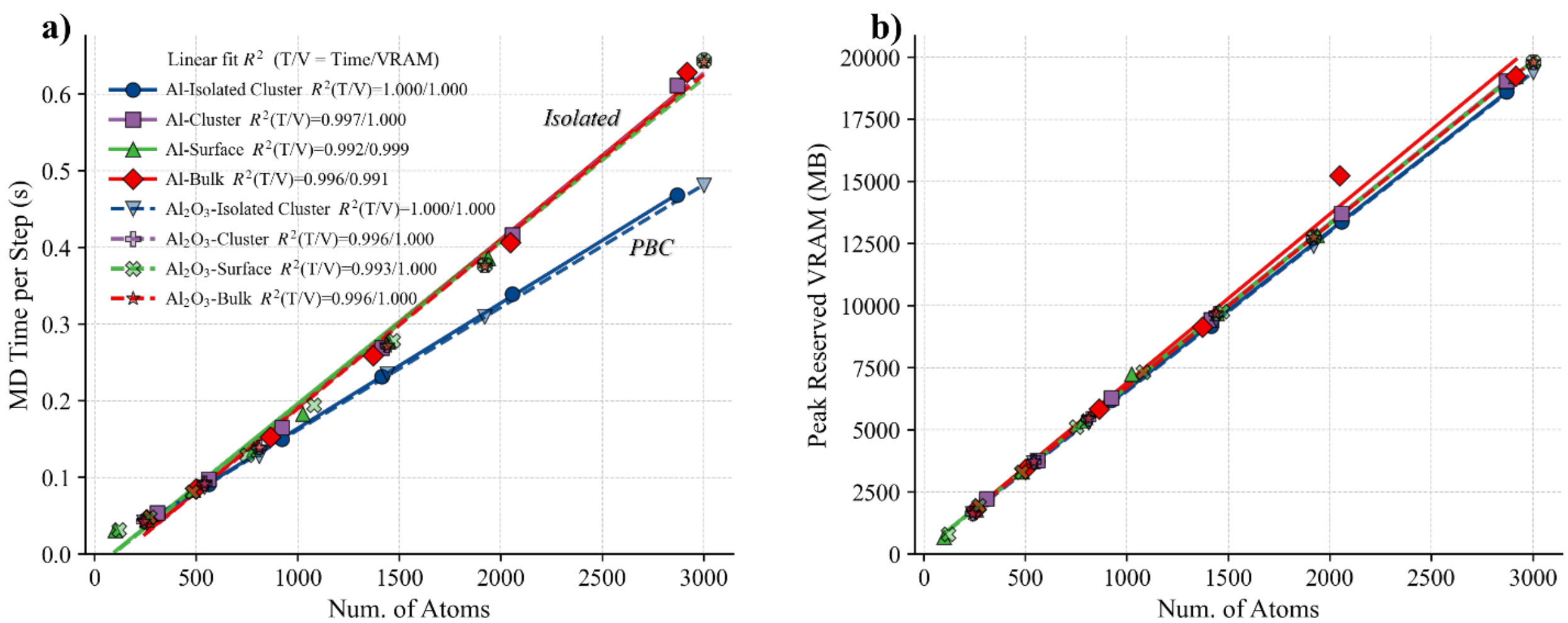


**Fig. S7.** MD inference benchmark for the trained MLP model. (a) Average wall time per MD step and (b) PyTorch peak reserved VRAM are plotted against the actual atom count for representative Al and $Al_2O_3$ bulk, finite-cluster, cluster-in-periodic-box, and surface/slab structures.

We quantified the inference cost of the trained model by benchmarking its MD simulation efficiency and GPU memory usage (Fig. S7). Simulations were performed using the Atomic Simulation Environment (ASE) [15] coupled with the DeePMD calculator. The setup included a Maxwell-Boltzmann velocity initialization at 300 K, a 1.0 fs timestep, 10 warm-up steps, and 50 timed steps for measurement. GPU memory consumption was tracked *via* PyTorch statistics. The test set comprised 52 representative Al and $Al_2O_3$ structures—including bulk, finite clusters, clusters in periodic boxes, and surface/slab models—with sizes ranging from 100 to 3000 atoms.

The benchmark shows that the average wall time ranged from 0.0312 to 0.644 seconds per MD step, while the peak reserved memory varied between 692 and 19812 MB. Across all tested structural classes, both the computational time per step and the reserved VRAM scaled approximately linearly with the atom count. These results demonstrate that the trained MLP model achieves efficient MD inference on a RTX 4090 GPU for systems up to 3000 atoms. Furthermore, the local-neighbor inference mechanism employed in the LAMMPS [16] pathway supports domain decomposition, enabling parallel execution for larger-scale, cross-node MD deployments.

## Section 3: Scientific-Agent Context and Human-Audited MLP Copilot Workflow

Table S3 organizes representative prior scientific-agent systems cited in the main text, together with additional related frameworks, according to their application domains and principal scientific roles. Because these systems address different tasks and were evaluated under different conditions, the table is intended as a descriptive overview rather than a quantitative performance comparison.

**Table S3** Categories of representative scientific-agent systems and AI-assisted scientific workflows

| Category | Representative systems | Primary domain and scope |
|---|---|---|
| General scientific research automation | The AI Scientist [17]; NovelSeek/InternAgent [18]; PaperQA [19]; Kosmos [20]; DORA [21] | Literature retrieval, hypothesis generation, code-based experiments, data analysis, and scientific reporting. |
| Chemistry reasoning and laboratory automation | ChemCrow [22]; Coscientist [23]; ORGANA [24]; ChemAgent [25] | Tool-assisted chemical reasoning, experimental planning, robotic execution, reaction optimization, and reusable chemical memory. |
| Domain-specific scientific reasoning and instrumentation | SciExplorer [26]; Ax-Prover [27]; StarWhisper Telescope [28] | Physical-model exploration, formal theorem proving, and automated astronomical observation and data processing. |
| Atomistic and computational science workflows | MDCrow [29]; El Agente [30]; El Agente Sólido [31]; El Agente Gráfico [32]; DREAMS [33]; ChemGraph [34] | Coordination of molecular dynamics, quantum chemistry, solid-state simulation, DFT/HPC execution, error recovery, and computational analysis. |
| Data-driven materials discovery and supporting infrastructure | Digital Catalysis Platform [35]; DIVE/DigHyd Agent [36]; StableOx-Cat [37]; Catalysis AI Agent [38]; Divalent Hydride AI Framework [39] | Scientific-data infrastructure, multimodal literature extraction, database-grounded screening, descriptor development, candidate generation, and physics-based validation. |

*Note: The scope column summarizes the range of capabilities represented across each category and does not imply that every listed system supports every capability.

Against this broader context, the coordination task in reactive-MLP development extends beyond any single calculation or simulation to an evolving lifecycle in which configurations, DFT labels, training datasets, model checkpoints, validation results, and intended model use must remain traceably connected. The emphasis of MLP Copilot is therefore not general scientific autonomy, but traceable, human-audited coordination of this lifecycle.

## 3.1 Role in Active-Learning MLP Development

In the ANP oxidation campaign, MLP Copilot implemented this lifecycle-oriented design as the coordination and audit layer for active-learning MLP development (Fig. S8). It linked the literature-derived project context, configuration datasets, DFT-labeling records, active-learning status, model checkpoints, validation outputs, and retained run artifacts.

Within this workflow, language-model agents assisted with task planning, evidence retrieval, validation-plan preparation, and report drafting. Numerical metrics, model predictions, plots, and validation results were generated by explicit computational tools and retained as auditable artifacts rather than inferred from language-model responses. DFT settings, training decisions, acceptance criteria, checkpoint selection, downstream model use, and scientific interpretation remained under expert control.

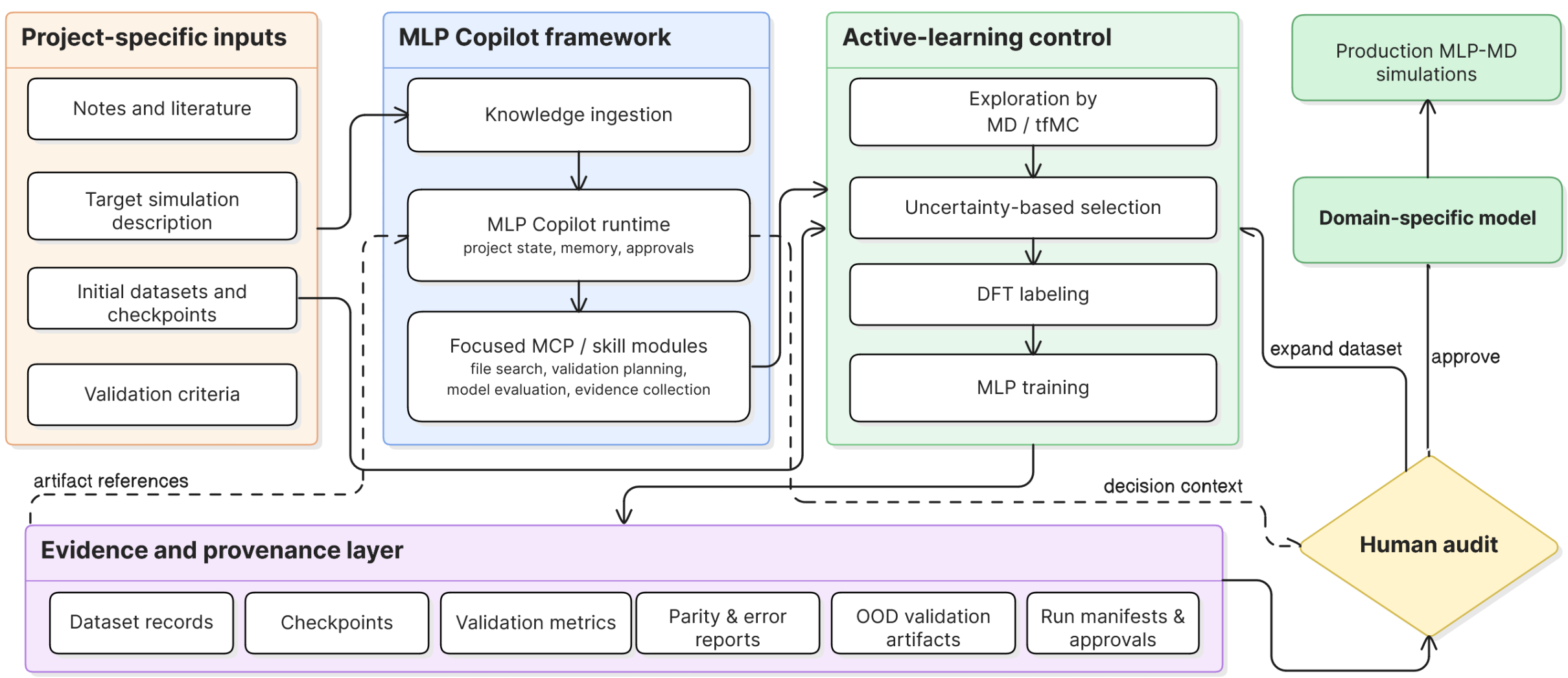


**Fig. S8.** Human-audited MLP Copilot framework used to coordinate task-specific MLP development and validation for the Al/O combustion system. The workflow links project-context retrieval, path-based tool access, artifact and run provenance, validation planning, and approval-gated decision support.

## 3.2 Evidence Flow, Provenance, and Approval Gates

Within this lifecycle, each MLP Copilot request followed an auditable evidence path. The researcher first specified the task goal and constraints, such as checking a checkpoint, inspecting an active-learning stage, or preparing a validation plan. MLP Copilot then retrieved relevant project context, selected an appropriate tool or workflow skill, and passed structures, datasets, checkpoints, or reports to tools by file path or artifact identifier. Tool execution produced metrics, plots, warnings, logs, reports, and prediction files in the workspace. These outputs were registered with run manifests and artifact records for later inspection. The practical implementation components of the MLP Copilot framework are summarized in Table S4.

Resource-intensive actions, such as DFT labeling and MLP training, and other state-changing actions were routed through approval gates, including launching or controlling active-

learning jobs, modifying training inputs, using a checkpoint for downstream simulation, or updating persistent project memory. The approval records preserve the requested action, decision state, reviewer, time stamp, and related run context.

Data management followed the same provenance principle. Short-term session context supported the current interaction, workspace/project state stored active project records, and artifact/run records preserved metrics, reports, figures, logs, hashes, and approval decisions. Large scientific payloads, including structures, trajectories, datasets, checkpoints, and force arrays, were retained as files or artifact references. The separation between the general workflow infrastructure and the domain-specific payloads (such as the trained potentials and ANP reference data) ensures that the core framework remains readily reusable for other active-learning-based MLP studies.

**Table S4** Practical implementation components of the MLP Copilot framework

| **Component** | **Role in the workflow** |
|---|---|
| Runtime and workspace state | Maintains the MLP-specific session, project records, dataset paths, checkpoint lineage, validation outputs, run records, approvals, logs, reports, figures, and artifacts. |
| Curated context search | Retrieves literature-derived and project-specific context for planning and drafting. Numerical validation is recorded through model-evaluation and dataset artifacts. |
| MCP tool packages | Provide DP-GEN active-learning inspection and control, dataset layout and integrity checks, checkpoint evaluation, trained-MLP inference, evidence-report generation, and local file search. |
| Scientific data access | Passes structures, trajectories, datasets, checkpoints, and reports by file path or artifact identifier, keeping large coordinate arrays and trajectories outside the language-model context. |
| Model evaluation tools | Generate checkpoint metrics, prediction artifacts, energy/force outputs, parity plots, and benchmark reports from explicit datasets and model files. |
| Artifact and run provenance | Records inputs, outputs, metrics, warnings, errors, lineage, hashes, approval links, and report artifacts in retained run manifests. |
| Approval gateway | Records approval decisions for resource-intensive, state-changing, workflow-control, or model-use actions. |
| Workflow skills | Provide task-specific guidance for active learning, machine-file preparation, dataset validation, checkpoint evaluation, validation planning, and OOD-test planning. |

### 3.3 Tool Demonstrations and Data Provenance

Figure S9 provides a representative terminal-workbench demonstration of the MLP Copilot workflow used for active-learning projects. In the task panel, the Copilot summarizes the project context, reports the current DP-GEN iteration and stage, identifies a blocking configuration issue, and points to the relevant project files and logs. The companion status and tool panels display the active run state, selected/accepted/failed frame counts, queue status, detected risks, tool-call records, DP-GEN artifacts, and approval entries. The demonstrated evidence flow reflects the data-provenance policy used in this work. Structures, trajectories, datasets, checkpoints, logs, reports, and figures are retained as files or artifact identifiers. Tool outputs are recorded with run/artifact metadata, including paths, hashes where available, metrics, warnings, errors, lineage, and approval links. This design allows the Copilot to coordinate literature/project retrieval, active-learning inspection, checkpoint evaluation, validation planning, and report preparation without treating the language-model response itself as the source of numerical scientific evidence. The source code is available online at github.com/flarecentury/mlpcopilot.

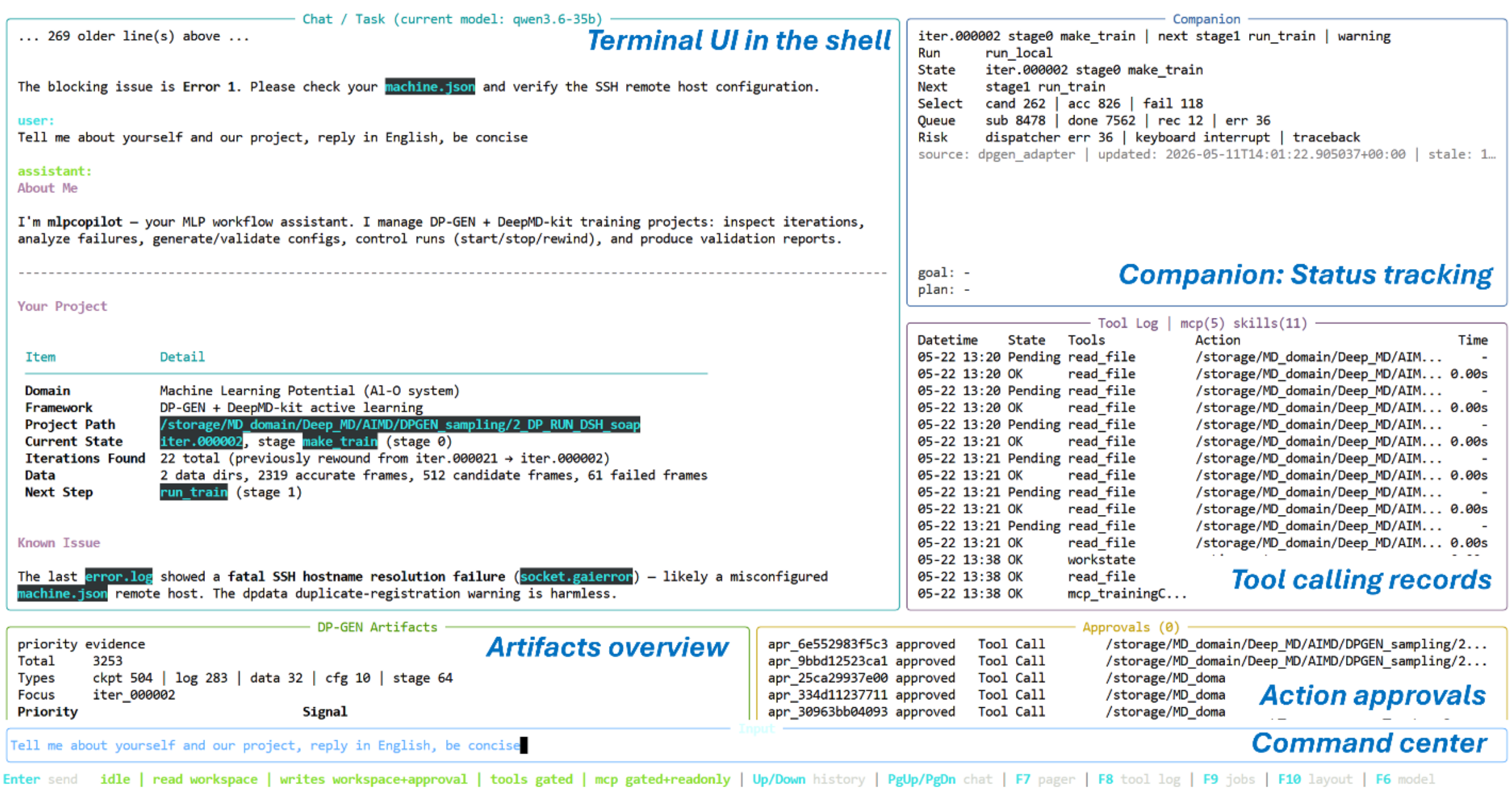


**Fig. S9.** Terminal UI demonstration of the MLP Copilot workflow for active-learning based MLP development. The screenshot shows project-context retrieval, current-state tracking, tool-call provenance, artifact indexing, and approval records in one human-audited interface. Scientific data and model files are referenced through paths or artifact records, while numerical validation and model-use decisions remain tied to retained tool outputs and human review.

## Section 4: Supplementary Results and Analysis

### 4.1 Gaussian-Density Analysis of Nanoparticle Morphology

We developed a novel approach for analyzing cluster morphology evolution using 2D Gaussian density field calculations with periodic boundary condition handling and parallel computation. The methodology employs the freud [40] computational framework to generate high-resolution density profiles that reveal the spatial distribution and clustering behavior of atomic species during combustion.

The Gaussian density calculation is formulated as:

$$\rho(\mathbf{r}) = \sum_{i=1}^{N} \frac{1}{(2\pi\sigma^2)^{3/2}} \exp\left(-\frac{|\mathbf{r}-\mathbf{r}_i|^2}{2\sigma^2}\right)$$

where $\sigma$ = 1Å represents the Gaussian smearing parameter, optimized for aluminum nanoparticle systems. The technique provides quantitative metrics for characterizing structural evolution through a systematic grid-based approach with resolution $D \times D$ (typically 10×10 bins) over analysis volumes of (50×50×50 $Å^3$). By mapping atomic positions onto continuous density fields, this methodology enables quantitative tracking of cluster formation, growth, and fragmentation processes central to understanding aluminum nanoparticle combustion mechanisms.

The implementation incorporates ASE for center-of-mass calculations with periodic boundary corrections, ensuring accurate density field generation even for clusters spanning simulation cell boundaries. The parallel processing strategy utilizes multiprocessing pools to efficiently compute density profiles across extensive trajectory datasets.

### 4.2 Oxygen-Migration Free-Energy Calculation

Figure S10 presents the free energy surface for oxygen hopping between aluminum bridge sites on the Al(100) surface, calculated using metadynamics simulations. The migration barrier of 1.25 eV explains the limited oxygen mobility at low temperatures and supports the proposed island-growth mechanism. The meta-dynamics simulations for oxygen migration barriers employ the PLUMED 2.8 [41] plugin interfaced with LAMMPS. The collective variable (CV) is defined as the distance between the migrating oxygen atom and the midpoint of two surface aluminum atoms, chosen to represent the migration pathway between adjacent bridge sites. The On-the-fly Probability Enhanced Sampling (OPES) method is used with adaptive Gaussian kernels [42], starting with an initial width of 0.1 Å and minimum width of 0.001 Å. The bias potential is deposited every 500 MD steps with a barrier height of 150 kJ/mol. Convergence is assessed by monitoring the evolution of the free energy profile, with simulations continued until the profile remains stable for at least 50 ps.

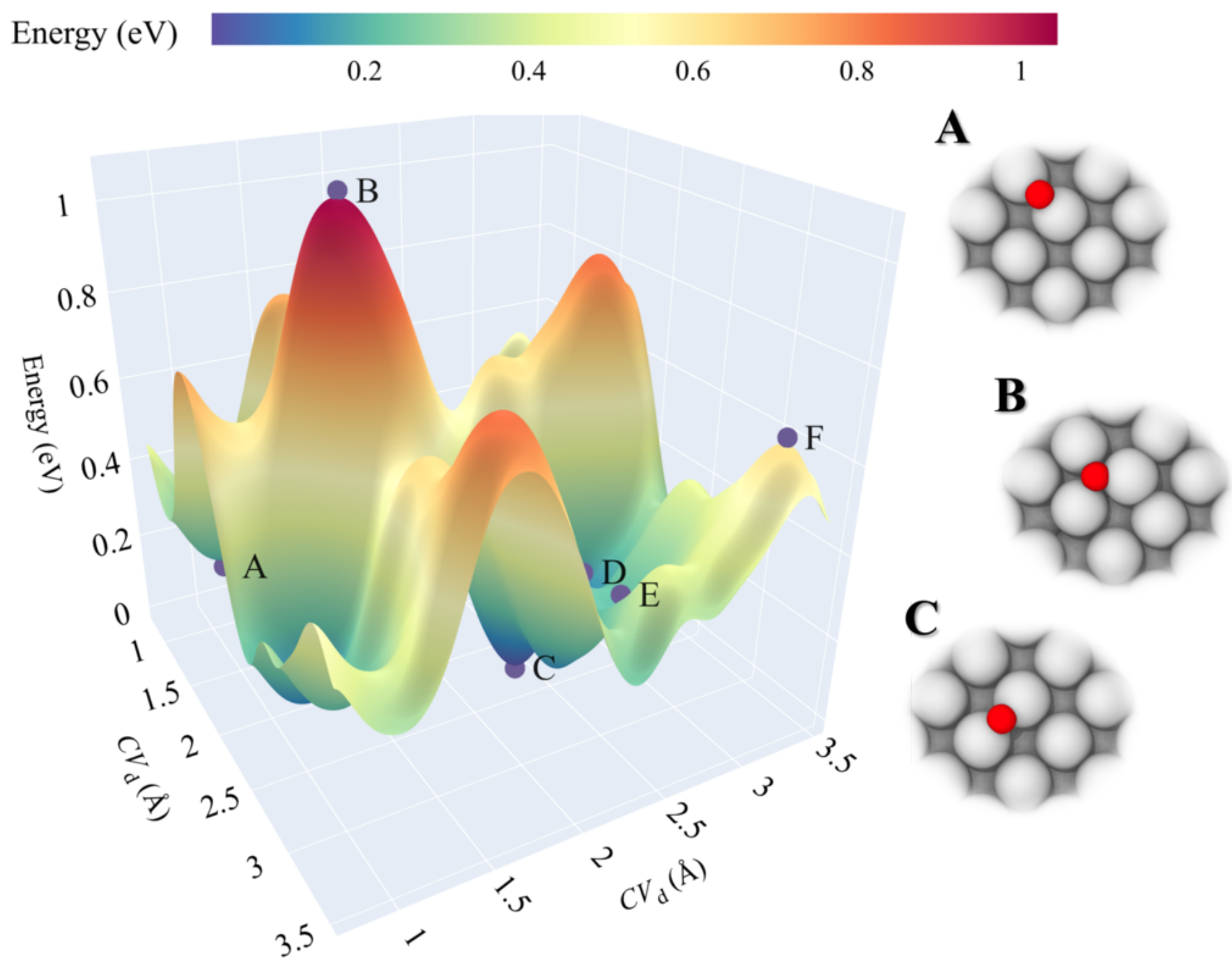


**Fig. S10.** Free energy surface for oxygen hopping between aluminum bridge sites.

## Section 5: Supplementary Figures

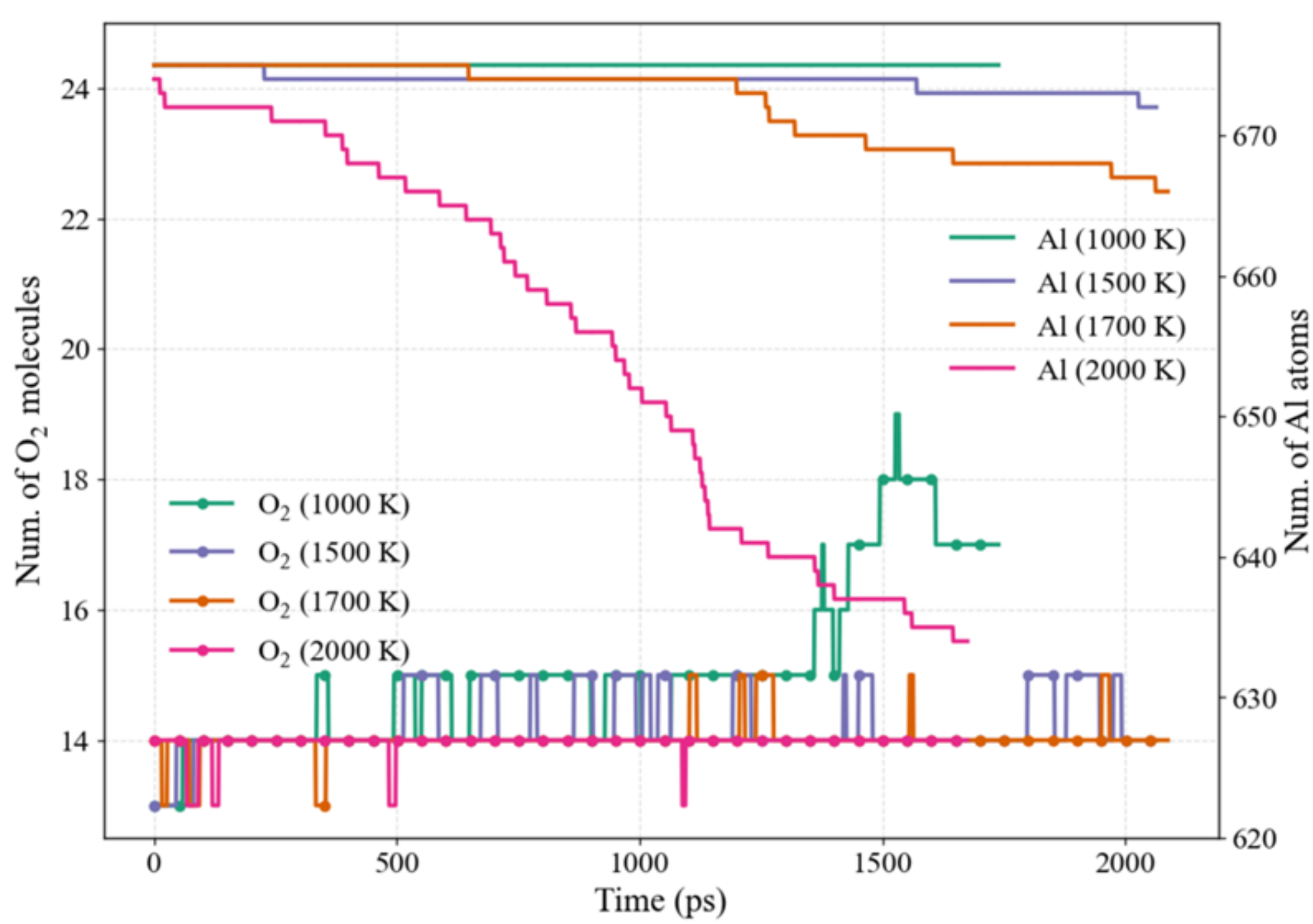


**Fig. S11.** Variation of $O_2$ molecule and Al atom numbers in the ANP bare metal over time at different temperatures.

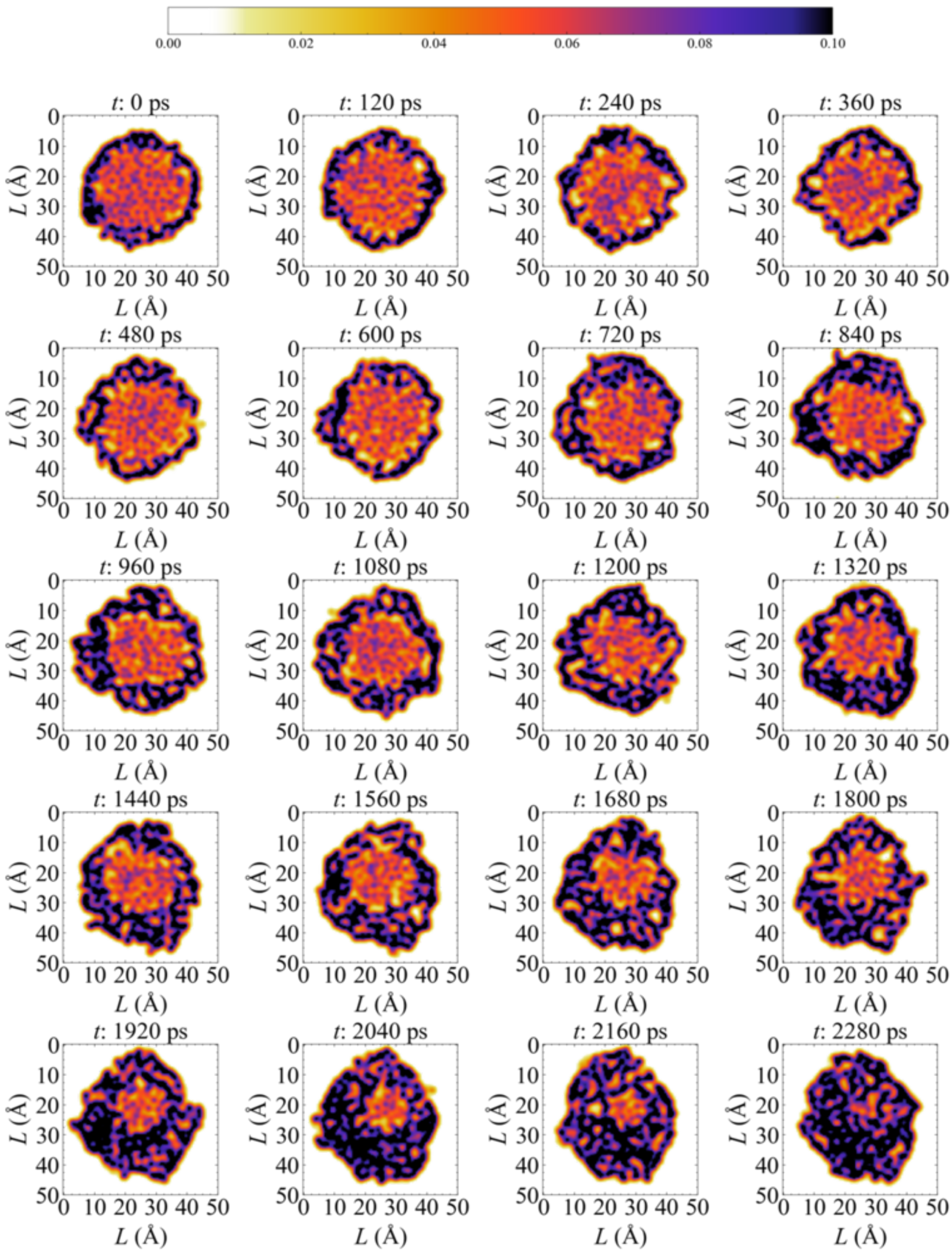


**Fig. S12.** Cross-sectional view of ANP particles during combustion at different temperatures (with shell, 1500 K).

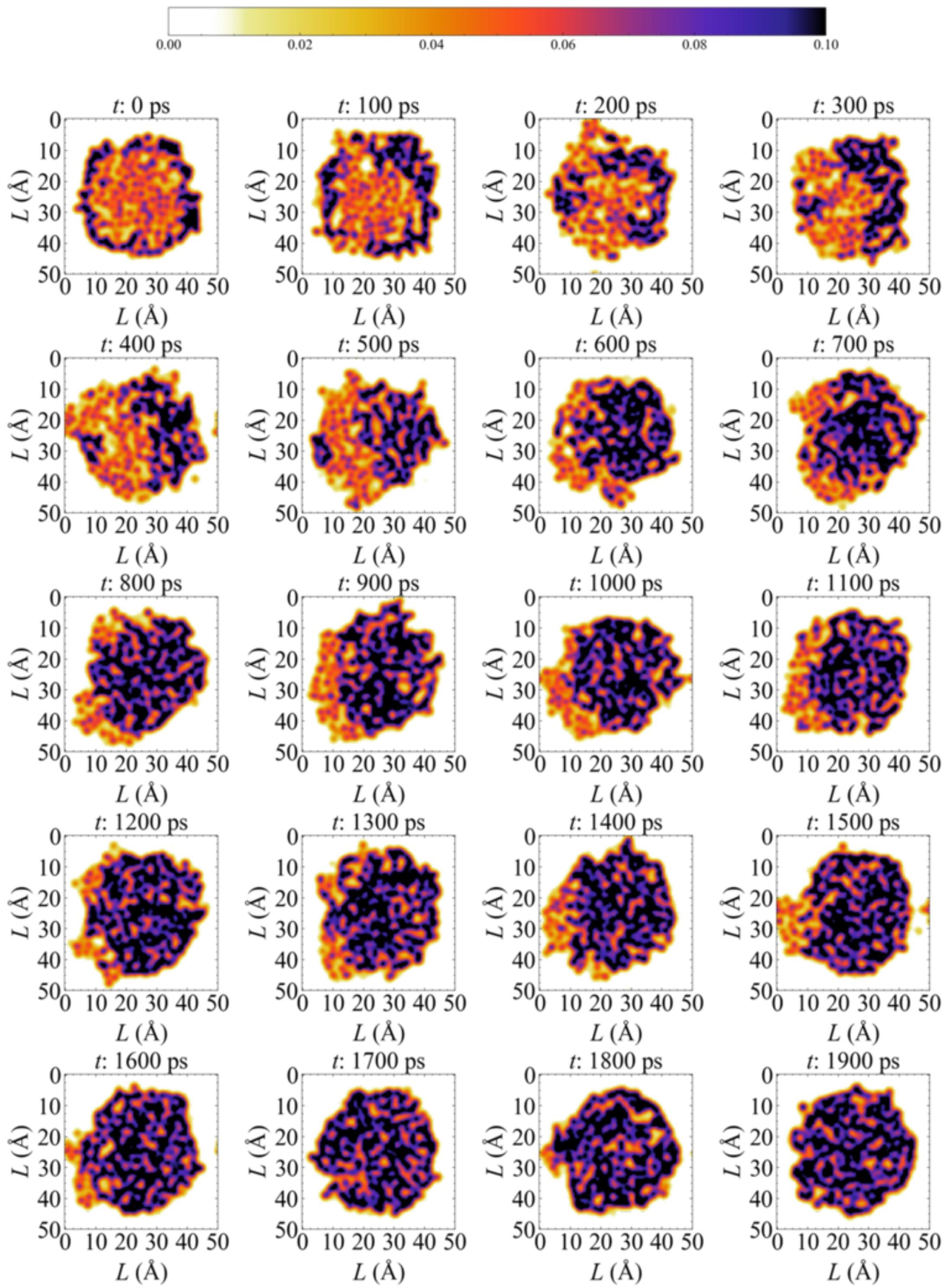

**Fig. S13.** Cross-sectional view of ANP particles during combustion at different temperatures (with shell, 2000 K).

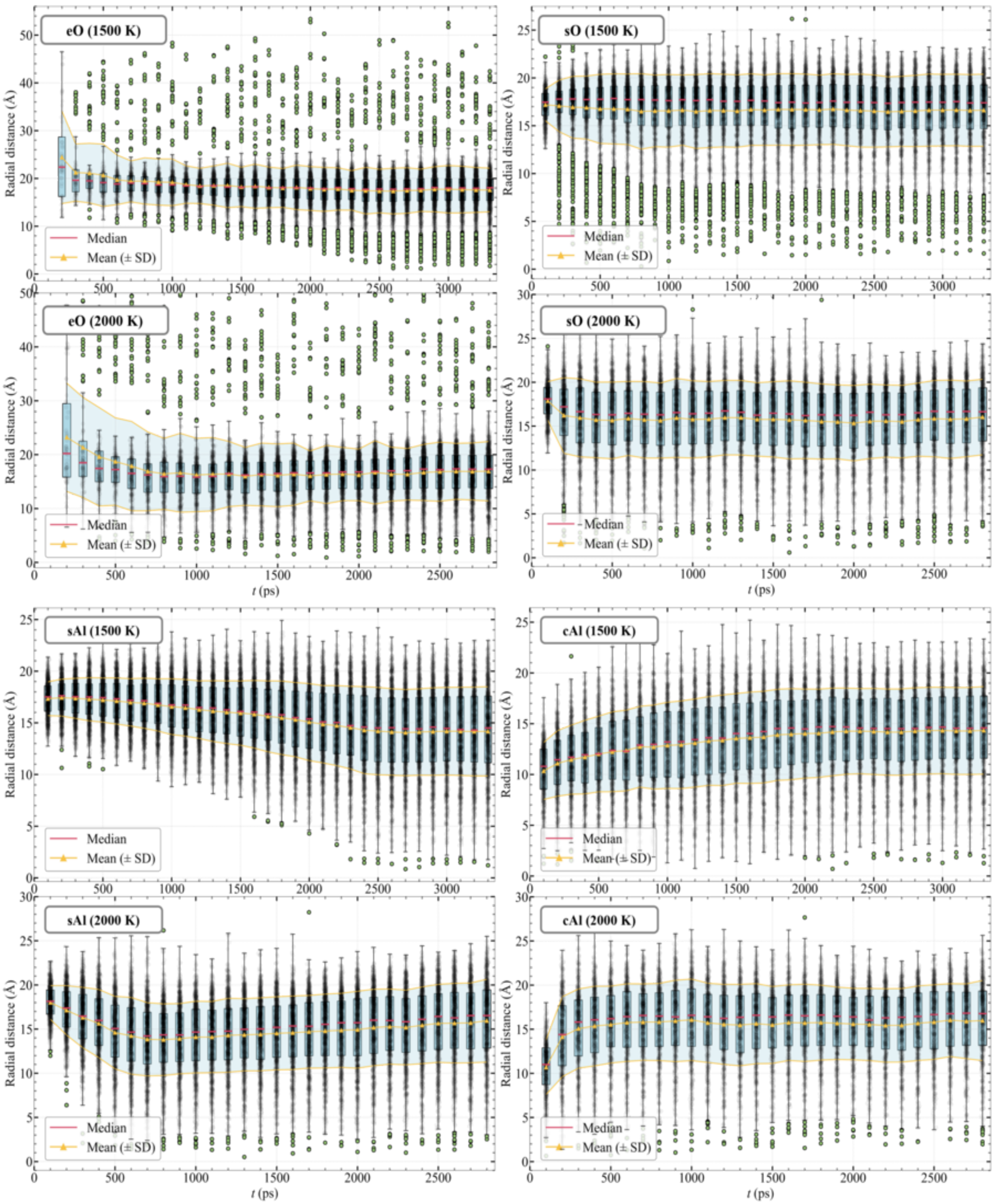


**Fig. S14.** Radial distance distribution of core aluminum (cAl), surface aluminum (sAl), and surface oxygen (sO) at 1500 K and 2000 K, illustrating the structural changes that occur during the transition from breathing mode to shell rupture mode.

## Section 6: SI Movie Legends

**Movie S1 (separate file).** Demonstration of the DigAuto platform and the multi-agent MLP Copilot workflow. The video showcases the human-audited workflow in which the MLP Copilot supports MLP quality auditing through validation tools and returns evidence for human expert review, enabling a closed-loop feedback mechanism for efficient model development.

**Movie S2 (separate file).** Molecular dynamics simulations of the oxidation process of a bare metal aluminum nanoparticle (ANP) at 1000 K and 1700 K.

**Movie S3 (separate file).** Molecular dynamics simulations of the combustion process of a core-shell aluminum nanoparticle (Al core with oxide shell) at 1500 K and 2000 K. The video demonstrates the distinct combustion behaviors, capturing the transition from breathing-like oxidation expansion to shell rupture as the temperature increases.

## References


[1] K. M. Bal and E. C. Neyts, "On the time scale associated with Monte Carlo simulations," *J. Chem. Phys.*, vol. 141, no. 20, p. 204104, Nov. 2014, doi: 10.1063/1.4902136.

[2] M. J. Mees, G. Pourtois, E. C. Neyts, B. J. Thijsse, and A. Stesmans, "Uniform-acceptance force-bias Monte Carlo method with time scale to study solid-state diffusion," *Phys. Rev. B*, vol. 85, no. 13, p. 134301, Apr. 2012, doi: 10.1103/PhysRevB.85.134301.

[3] Y. Zhang *et al.*, "DP-GEN: A concurrent learning platform for the generation of reliable deep learning based potential energy models," *Computer Physics Communications*, vol. 253, p. 107206, Aug. 2020, doi: 10.1016/j.cpc.2020.107206.

[4] D. Zhang, P. Yi, X. Lai, L. Peng, and H. Li, "Active machine learning model for the dynamic simulation and growth mechanisms of carbon on metal surface," *Nat Commun*, vol. 15, no. 1, Art. no. 1, Jan. 2024, doi: 10.1038/s41467-023-44525-z.

[5] B. Cheng *et al.*, "Mapping Materials and Molecules," *Acc. Chem. Res.*, vol. 53, no. 9, pp. 1981–1991, Sep. 2020, doi: 10.1021/acs.accounts.0c00403.

[6] H. Wang, L. Zhang, J. Han, and W. E, "DeePMD-kit: A deep learning package for many-body potential energy representation and molecular dynamics," *Computer Physics Communications*, vol. 228, pp. 178–184, Jul. 2018, doi: 10.1016/j.cpc.2018.03.016.

[7] D. Zhang *et al.*, "DPA-2: a large atomic model as a multi-task learner," *npj Comput Mater*, vol. 10, no. 1, p. 293, Dec. 2024, doi: 10.1038/s41524-024-01493-2.

[8] F. H. Streitz and J. W. Mintmire, "Electrostatic potentials for metal-oxide surfaces and interfaces," *Phys. Rev. B*, vol. 50, no. 16, pp. 11996–12003, Oct. 1994, doi: 10.1103/PhysRevB.50.11996.

[9] S. Hong and A. C. T. van Duin, "Atomistic-Scale Analysis of Carbon Coating and Its Effect on the Oxidation of Aluminum Nanoparticles by ReaxFF-Molecular Dynamics Simulations," *J. Phys. Chem. C*, vol. 120, no. 17, pp. 9464–9474, May 2016, doi: 10.1021/acs.jpcc.6b00786.

[10] I. Kruglov, O. Sergeev, A. Yanilkin, and A. R. Oganov, "Energy-free machine learning force field for aluminum," *Sci Rep*, vol. 7, no. 1, p. 8512, Aug. 2017, doi: 10.1038/s41598-017-08455-3.

[11] Y.C. Wu and J.L. Shao, "mdapy: A flexible and efficient analysis software for molecular dynamics simulations," *Computer Physics Communications*, vol. 290, p. 108764, Sep. 2023, doi: 10.1016/j.cpc.2023.108764.

[12] J. S. Smith *et al.*, "Automated discovery of a robust interatomic potential for aluminum," *Nat Commun*, vol. 12, no. 1, p. 1257, Feb. 2021, doi: 10.1038/s41467-021-21376-0.

[13] D. Alimonti and F. Baletto, "Machine-learnt potential highlights melting and freezing of aluminum nanoparticles," *J. Chem. Phys.*, vol. 162, no. 14, p. 144302, Apr. 2025, doi: 10.1063/5.0253649.

[14] L. Chaput, A. Togo, and I. Tanaka, "Finite-displacement computation of the electron-phonon interaction within the projector augmented-wave method," *Phys. Rev. B*, vol. 100, no. 17, p. 174304, Nov. 2019, doi: 10.1103/PhysRevB.100.174304.

[15] A. H. Larsen *et al.*, "The atomic simulation environment—a Python library for working with atoms," *J Phys-condens Mat*, vol. 29, no. 27, p. 273002, Jun. 2017, doi: 10.1088/1361-648X/aa680e.

[16] A. P. Thompson *et al.*, "LAMMPS - a flexible simulation tool for particle-based materials modeling at the atomic, meso, and continuum scales," *Comput Phys Commun*, vol. 271, p. 108171, Feb. 2022, doi: 10.1016/j.cpc.2021.108171.

[17] C. Lu *et al.*, "Towards end-to-end automation of AI research," *Nature*, vol. 651, no. 8107, pp. 914–919, Mar. 2026, doi: 10.1038/s41586-026-10265-5.

[18] I. Team *et al.*, "InternAgent: When Agent Becomes the Scientist – Building Closed-Loop System from Hypothesis to Verification," 2025. [Online]. Available: https://arxiv.org/abs/2505.16938

[19] J. Lála, O. O'Donoghue, A. Shtedritski, S. Cox, S. G. Rodriques, and A. D. White, "PaperQA: Retrieval-Augmented Generative Agent for Scientific Research," arXiv.org. Accessed: Jul. 23, 2026. [Online]. Available: https://arxiv.org/abs/2312.07559v2

[20] L. Mitchener *et al.*, "Kosmos: An AI Scientist for Autonomous Discovery," arXiv.org. Accessed: Jul. 23, 2026. [Online]. Available: https://arxiv.org/abs/2511.02824v2

[21] V. Naumov *et al.*, "DORA AI Scientist: Multi-agent Virtual Research Team for Scientific Exploration Discovery and Automated Report Generation," *bioRxiv*, 2025, doi: 10.1101/2025.03.06.641840.

[22] A. M. Bran, S. Cox, O. Schilter, C. Baldassari, A. D. White, and P. Schwaller, "Augmenting large language models with chemistry tools," *Nat Mach Intell*, vol. 6, no. 5, pp. 525–535, May 2024, doi: 10.1038/s42256-024-00832-8.

[23] D. A. Boiko, R. MacKnight, B. Kline, and G. Gomes, "Autonomous chemical research with large language models," *Nature*, vol. 624, no. 7992, pp. 570–578, Dec. 2023, doi: 10.1038/s41586-023-06792-0.

[24] K. Darvish *et al.*, "ORGANA: A robotic assistant for automated chemistry experimentation and characterization," *Matter*, vol. 8, no. 2, Feb. 2025, doi: 10.1016/j.matt.2024.10.015.

[25] X. Tang *et al.*, "ChemAgent: Self-updating Memories in Large Language Models Improves Chemical Reasoning," *International Conference on Learning Representations*, vol. 2025, pp. 100922–100958, May 2025.

[26] M. Nägele and F. Marquardt, "Agentic Exploration of Physics Models," *Phys. Rev. X*, vol. 16, no. 3, p. 031002, Jul. 2026, doi: 10.1103/xnqc-q6nt.

[27] B. Breen *et al.*, "Ax-Prover: A Deep Reasoning Agentic Framework for Theorem Proving in Mathematics and Quantum Physics," arXiv.org. Accessed: Jul. 23, 2026. [Online]. Available: https://arxiv.org/abs/2510.12787v4

[28] C. Wang *et al.*, “StarWhisper Telescope: an AI framework for automating end-to-end astronomical observations,” *Commun Eng*, vol. 4, no. 1, p. 184, Nov. 2025, doi: 10.1038/s44172-025-00520-4.
[29] Q. Campbell, S. Cox, J. Medina, B. Watterson, and A. D. White, “MDCrow: automating molecular dynamics workflows with large language models,” *Mach. Learn.: Sci. Technol.*, vol. 7, no. 2, p. 025037, Mar. 2026, doi: 10.1088/2632-2153/ae4b07.
[30] Y. Zou *et al.*, “El Agente: An autonomous agent for quantum chemistry,” *Matter*, vol. 8, no. 7, Jul. 2025, doi: 10.1016/j.matt.2025.102263.
[31] S. G. H. Kumar *et al.*, “El Agente Sólido: A New Age(nt) for Solid State Simulations,” 2026. [Online]. Available: https://arxiv.org/abs/2602.17886
[32] J. Bai *et al.*, “El Agente Gráfico: Structured Execution Graph for Scientific Agents,” presented at the AI4X {\textendash} Accelerate Conference 2026, Mar. 2026. Accessed: Jul. 23, 2026. [Online]. Available: https://openreview.net/forum?id=YShauPAyTw
[33] Z. Wang *et al.*, “DREAMS: Density Functional Theory Based Research Engine for Agentic Materials Simulation,” arXiv.org. Accessed: Jul. 23, 2026. [Online]. Available: https://arxiv.org/abs/2507.14267v1
[34] T. D. Pham, A. Tanikanti, and M. Keçeli, “ChemGraph as an agentic framework for computational chemistry workflows,” *Commun Chem*, vol. 9, no. 1, p. 33, Jan. 2026, doi: 10.1038/s42004-025-01776-9.
[35] D. Zhang *et al.*, “Digital catalysis platform as a gateway to big data and AI-powered innovations in catalysis,” *Chem Catalysis*, vol. 6, no. 7, p. 101775, Jul. 2026, doi: 10.1016/j.checat.2026.101775.
[36] D. Zhang *et al.*, “‘DIVE’ into hydrogen storage materials discovery with AI agents,” *Chem. Sci.*, vol. 17, no. 6, pp. 3031–3042, Feb. 2026, doi: 10.1039/d5sc09921h.
[37] “StableOx-Cat agent: an AI agent for exploring stable metal oxide electrocatalysts,” *aiagent*, vol. 2, no. 1, p. N/A-N/A, Mar. 2026, doi: 10.20517/aiagent.2026.03.
[38] X. Wang, Z. Li, D. Zhang, H. Li, H. Xu, and D. Cheng, “Catalysis AI Agent Guides Discovering the Universal Design Principle of Cu-Based Single-Atom Alloy Catalysts for CO2 Electroreduction,” *Angewandte Chemie International Edition*, vol. 65, no. 21, p. e24612, 2026, doi: 10.1002/anie.202524612.
[39] Q. Wang *et al.*, “Unraveling the Complexity of Divalent Hydride Electrolytes in Solid-State Batteries via a Data-Driven Framework with Large Language Model,” *Angewandte Chemie International Edition*, vol. 64, no. 25, p. e202506573, 2025, doi: 10.1002/anie.202506573.
[40] V. Ramasubramani, B. D. Dice, E. S. Harper, M. P. Spellings, J. A. Anderson, and S. C. Glotzer, “freud: A software suite for high throughput analysis of particle simulation data,” *Computer Physics Communications*, vol. 254, p. 107275, Sep. 2020, doi: 10.1016/j.cpc.2020.107275.
[41] M. Bonomi *et al.*, “Promoting transparency and reproducibility in enhanced molecular simulations,” *Nat Methods*, vol. 16, no. 8, pp. 670–673, Aug. 2019, doi: 10.1038/s41592-019-0506-8.
[42] M. Invernizzi and M. Parrinello, “Rethinking Metadynamics: From Bias Potentials to Probability Distributions,” *J. Phys. Chem. Lett.*, vol. 11, no. 7, pp. 2731–2736, Apr. 2020, doi: 10.1021/acs.jpclett.0c00497.
